# An ultra-stable cytoplasmic antibody engineered for in vivo applications

**DOI:** 10.1038/s41467-019-13654-9

**Published:** 2020-01-17

**Authors:** Hiroyuki Kabayama, Makoto Takeuchi, Naoko Tokushige, Shin-ichi Muramatsu, Miyuki Kabayama, Mitsunori Fukuda, Yoshiyuki Yamada, Katsuhiko Mikoshiba

**Affiliations:** 1 Laboratory for Developmental Neurobiology, Center for Brain Science, Institute of Physical and Chemical Research, 2-1 Hirosawa, Wako, Saitama 351-0198 Japan; 2 Laboratory of Veterinary Biochemistry, School of Veterinary Medicine, Nippon Veterinary and Life Science University, Tokyo, 1-7-1 Kyonantyo, Musashino, Tokyo 180-8602 Japan; 3 Division of Neurological Gene Therapy, Center for Open Innovation, Jichi Medical University, 3311-1 Yakushiji, Shimotsuke, Tochigi 329-0498 Japan; 4 Center for Gene & Cell Therapy, The Institute of Medical Science, The University of Tokyo, Tokyo, 108-8639 Japan; 5 Division of Functional Morphology, Department of Basic Veterinary Medicine, School of Veterinary Medicine, Nippon Veterinary and Life Science University, 1-7-1 Kyonantyo, Musashino, Tokyo 180-8602 Japan; 6 Laboratory of Membrane Trafficking Mechanisms, Department of Integrative Life Sciences, Graduate School of Life Sciences, Tohoku University, Aobayama, Aoba-ku, Sendai, Miyagi 980-8578 Japan; 7 Shanghai Institute for Advanced Immunochemical Studies, ShanghaiTech University, Y building, 393 Middle Huaxia Road, Shanghai, 201210 China; 8 Faculty of Science, Toho University, 2-2-1 Miyama, Funabashi-shi, Chiba, Tokyo 274-8510 Japan

**Keywords:** Protein design, Applied immunology, Cancer therapy

## Abstract

Targeting cytoplasmic protein–protein interactions with antibodies remains technically challenging, since antibodies expressed in the cytosol frequently form insoluble aggregates. Existing engineering methods are based on the notion that the estimated net charge at pH 7.4 affects stability; as such, they are unable to overcome this problem. Herein, we report a versatile method for engineering an ultra-stable cytoplasmic antibody (STAND), with a strong estimated net negative charge at pH 6.6, by fusing peptide tags with a highly negative charge and a low isoelectric point. Without the need for complicated amino acid substitutions, we convert aggregation-prone antibodies to STANDs that are useful for inhibiting in vivo transmitter release, modulating animal behaviour, and inhibiting in vivo cancer proliferation driven by mutated Kras—long recognised as an “undruggable” oncogenic protein. The STAND method shows promise for targeting endogenous cytoplasmic proteins in basic biology and for developing future disease treatments.

## Abstract (web summary)

Antibodies expressed in the cytosol often form insoluble aggregates, which makes it hard to target intracellular proteins. Here the authors engineer an ultra-stable cytoplasmic antibody (STAND) with a low isoelectric point that can be used in vivo.

## Introduction

Antibodies are indispensable basic research tools and proven candidates for therapeutic development because they specifically bind to antigens and interfere with targeted molecular pathways by inhibiting protein–protein interactions. While many key drug targets are intracellular molecules, most antibody-based therapies are limited to extracellular targets^1^. One of the most frequently used intracellular antibody formats (intrabody) is the single-chain (sc) variable domain (Fv; termed scFv), in which variable domains of the heavy and the light chains of antibodies are connected via a flexible glycine linker (3 × GGGGS)^2^. Cytoplasmic expression of scFv is a long-standing challenge due to its tendency to misfold and aggregate; the cytoplasm is a reducing environment, where disulfide bridge formation within the Fvs of the light and heavy chains of scFv molecules can be prevented^3–6^. A previous study showed that ~0.1% cytoplasmic intrabodies among clones screened from a naive human-spleen-cell-derived scFv library were stable and functional^7^, highlighting the difficulty of obtaining the intrabodies.

Currently, there is no reliable method to engineer stable cytoplasmic intrabodies in mammalian cells. Screening of stable scFvs from hybridoma clones and/or introducing structural analysis-based amino acid substitutions is necessary to improve their folding and stability in the cytoplasm^8^. Thus, isolation of stable cytoplasmic intrabodies requires considerable time and effort. To overcome this, researchers have investigated intrabody formats—camelids-derived single-domain antibodies (VHHs), and antibody-like fibronectin-derived proteins (FingRs) that fold stably with no disulfide bonds^9,10^. Nevertheless, VHHs are not always stably expressed in the cytoplasm of cultured mammalian cells^4,11^; only 10−20% of cytoplasmic FingRs among clones screened from a FingR library were stable and functional^10^. Hence, engineering VHH or FingR proteins, or screening for more stable clones and scFv proteins, is necessary^10,12^.

A recent study suggested that the physicochemical parameters of intrabodies are important for developing stable cytoplasmic intrabodies; the intrinsic net charge of intrabodies at cytoplasmic pH 7.4 affects their aggregation propensity in the cytoplasm of cultured mammalian cells. Further, fusing highly negatively charged peptide tags to scFvs improves their solubility, possibly by increasing the net negative charge^4,12^. This approach is likely effective for reducing the probability of cytoplasmic aggregation of intrabodies; however, it has 3 major limitations: a low success rate in reducing intrabody aggregation, insufficient correlation between the estimated net negative charge of intrabodies at pH 7.4 with experimentally determined stability, and a lack of in vivo validation for the targeting of endogenous proteins^4^. In fact, using a negatively charged 3 × Flag (DYKDDDDK) peptide tag for scFv-D5—an aggregation-prone intrabody against amyloid oligomers—was found to decrease its aggregation; however, 10% of the cells still exhibited aggregates in the cytoplasm of cultured mammalian cells^4^. Additionally, intrabodies with similar net negative charges at pH 7.4 often have different aggregation propensities^4^. These problems hamper the rapid and easy development of stable cytoplasmic antibodies.

Here, we present a versatile method for generating an ultra-stable cytoplasmic antibody (STAND) for in vivo applications. We focused on the net charge of intrabodies at a broad pH range (6.6–7.4) and their isoelectric points (pIs), since cytosolic pH values change from 6.3–7.5 during development or in response to various stimuli^13–19^. These findings suggest the possibility of unexpectedly exposing cytoplasmic intrabodies to lower pH environments at the cytoplasm of cells in vivo. Proteins have a net positive charge in a pH below their pI, and a net negative charge in a pH above it. Thus, whether intrabodies can retain the strong net negative charge under a lower pH is dependent on their pI. We report that in silico calculation of the net charge of intrabodies at pH 6.6, but not pH 7.4, is critical for designating STANDs. The cytoplasmic antibodies with a low pI and strong net negative charge, even at pH 6.6, were designated STANDs; they are stable and act in the cytoplasm in vitro and in vivo.

## Results

### Engineering ultra-stable cytoplasmic antibodies (STANDs)

**Fig. 1:**
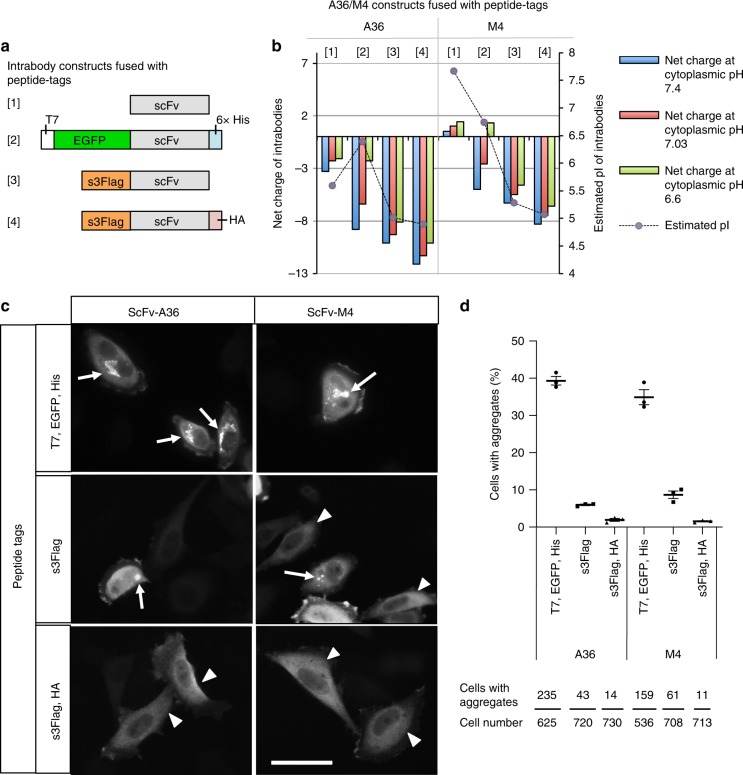
Engineering ultra-stable cytoplasmic antibodies (STANDs). **a** Schematic representation of scFv constructs fused with various peptide tags: [1] non-tagged scFv, [2] scFv with N-terminal T7 and EGFP, and C-terminal 6× His (scFv-GFP), [3] scFv with N-terminal s3Flag (s3Flag-scFv), [4] ScFv with N-terminal s3Flag and C-terminal HA (s3Flag-scFv-HA). **b** Net charge comparison of scFv-A36 and scFv-M4 proteins fused with indicated peptide tags at cytoplasmic pH 6.6 (green), 7.03 (red), and 7.4 (blue), and the isoelectric point (pI) determination (grey circle) using in silico physicochemical analysis. **c** Intracellular stability comparison of scFv-A36 (left panels) and scFv-M4 (right panels) fused with different peptide tags expressed in HeLa cells using immunocytochemistry; anti-Flag (lower and middle panels) or anti-T7 (upper panels) antibodies were used to detect scFv proteins. Arrows indicate intracellular scFv aggregates. Arrowheads indicate scFvs stably expressed in the cytoplasm. Scale bar, 50 μm. **d** Percentage of scFv-expressing cells with aggregates in **c** (upper panel) and total cell and aggregated cell number in 3 independent experiments (bottom panel). Statistical analysis: two-tailed one-way analysis of variance (A36; percentage of aggregation, *F* (2, 6) = 834.25, *P* < 0.00001; Tukey’s multiple comparison test, s3Flag-A36-HA vs. s3Flag-A36: *P* = 0.01614, s3Flag-A36-HA vs. scFv-GFPA36: *P* < 0.00001, s3Flag-A36 vs. scFv-GFPA36: *P* < 0.00001; M4; percentage of aggregation, *F* (2, 6) = 180.65, *P* < 0.00001; Tukey’s multiple comparison test, s3Flag-M4-HA vs. s3Flag-M4: *P* = 0.01949, s3Flag-M4-HA vs. scFv-GFPM4: *P* < 0.00001, s3Flag-M4 vs. scFv-GFPM4: *P* < 0.00001). Error bars represent standard error of the mean. Source data are provided as a Source Data file.

To determine the extent to which pI and pH values contribute to the net charge of intrabodies in silico, we first designed intrabody constructs with various pIs and net charges by fusing the intrabodies with different peptide tags (Fig. 1a) and investigated which peptide tags are appropriate for creating stable intrabodies. As scFv sequence models, we used a newly isolated scFv clone (termed scFv-A36) that specifically binds to the C2A domain of synaptotagmin I and II (Syt I/II-C2A), isoforms of the synaptotagmin family that regulate membrane trafficking, and its antigen binding activity-lacking mutant (termed scFv-M4) (Supplementary Fig. 1). Fusion of T7, enhanced green fluorescent protein (EGFP), and histidine tags to scFv-A36 or scFv-M4 (termed scFv-GFPA36 or scFv-GFPM4) increased the net negative charge of both proteins at pH 7.4 (Fig. 1a, b); however, the negative charge was markedly reduced at pH 7.03 (Fig. 1b) and 6.6 (Fig. 1b). The reduction of the net negative charge was due to their relatively high pI (Fig. 1b).

To increase the net negative charge of intrabodies at the lower pH, we tested another 3× Flag tag (DYKDHDGDYKDHDIDYKDDDDK, developed by Sigma, termed s3Flag to distinguish from 3 × Flag [DYKDDDDK]), which has a strong net negative charge (−7.0) and a low pI (4.48). As expected, an in silico analysis revealed that fusing an s3Flag tag to an scFv-A36 or scFv-M4 (termed s3Flag-scFv-A36 or s3Flag-scFv-M4) decreased their pI (Fig. 1a) and increased their net negative charge (Fig. 1b), even at pH 6.6. It has been reported that the hydrophobic surface of the bottom structure of the Fv region in heavy chains and light chains contributes to the aggregation of scFv^20^. We attempted to antagonise this effect on stability by further increasing the net negative charge of scFvs at a lower pH and by decreasing their pI through fusion of the human influenza hemagglutinin (HA) tag (YPYDVPDYA) with low pI (3.53) and a net negative charge (−2.2) to the C-terminus of s3Flag-scFv-A36 or s3Flag-scFv-M4 (Fig. 1a). As expected, fusing s3Flag and HA tags to scFv-A36 or scFv-M4 (termed s3Flag-scFv-A36-HA or s3Flag-scFv-M4-HA) further decreased their pI (Fig. 1b) while increasing their net negative charge (Fig. 1b), even at pH 6.6. Next, we tested the generalisability of the effects of these peptide tags on the net charge by using 94 other scFv proteins obtained from an NCBI BLAST search of the scFv-A36 sequence; we found that their effects were similar to those of scFv-A36 and scFv-M4 (Supplementary Fig. 2).

Afterwards, we compared the stability of scFv-A36 and scFv-M4 fused with these peptide tags in the cytoplasm of HeLa cells. An immunocytochemical analysis revealed that s3Flag-scFv-A36-HA and s3Flag-scFv-M4-HA were diffusely expressed in the cytoplasm (Fig. 1c, arrowheads), with little aggregation compared to other constructs, which formed aggregates (Fig. 1c, arrows). A quantitative analysis confirmed that the cells expressing scFv-GFP exhibited cytoplasmic aggregates (A36, 39.33 ± 0.94%; M4, 34.91 ± 1.65%; Fig. 1d). Although fusing an s3Flag tag alone to scFv-A36 or scFv-M4 was effective, the cells expressing these intrabodies exhibited aggregates (A36, 5.98 ± 0.17%; M4, 8.69 ± 0.82%). The average number of cells with cytoplasmic aggregates was significantly lower in cells expressing s3Flag-scFv-A36-HA (1.93 ± 0.31%) or s3Flag-scFv-M4-HA (1.55 ± 0.14%) than in those expressing s3Flag-scFv-A36 or s3Flag-scFv-M4 (Fig. 1d). We also noted that scFv-GFPA36, which has a slightly stronger net negative charge at pH 7.4 than s3Flag-scFv-M4-HA (Fig. 1b), exhibited a considerably higher percentage of aggregates than s3Flag-scFv-M4-HA (Fig. 1c, d). These different aggregation properties of intrabodies with similar net negative charges at pH 7.4 were consistent with the frequently observed properties of intrabodies previously reported^4^.

**Fig. 2:**
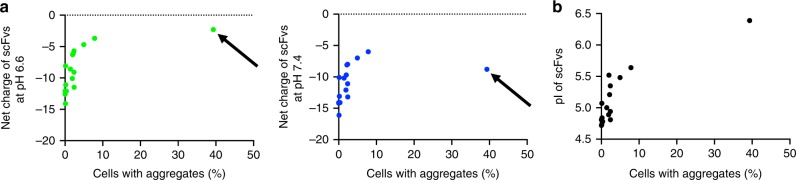
Correlated aggregation rate and net negative charge of scFvs at pH 6.6. **a** An additional 15 s3Flag-scFv-HA proteins derived from scFvs in supplementary Fig. 2 were constructed, and their aggregation rate in HeLa cells was quantified using immunocytochemical analysis. Statistical correlation analysis of the net negative charge and aggregation rate of 16 s3Flag-scFv-HA proteins including s3Flag-scFv-A36-HA, and scFv-GFPA36 in HeLa cells (17 scFvs were tested) (left, net negative charge at pH 6.6 vs. aggregation rate: *r* = 0.6168, *P* = 0.008179; right, net negative charge at pH 7.4 vs. aggregation rate: *r* = 0.3709, *P* = 0.1427). The arrow in left panel (pH 6.6) indicates a net charge value of scFv-GFPA36, which is shifted to a higher absolute value of the negative charge in the right panel (pH 7.4), owing to its relatively high pI. The success rate of stable expression (less than around 2% aggregation) of s3Flag-scFv-HA proteins (16 total proteins) was found to be ~82%. **b** Statistical correlation analysis of pIs and aggregation rates of 17 scFv proteins used in **a** (*r* = 0.8392, *P* = 0.000025). Source data are provided as a Source Data file.

Importantly, scFv-GFPA36, but not s3Flag-scFv-M4-HA, showed a marked reduction in the net negative charge at pH 6.6 (Fig. 1b), raising the hypothesis that the low pI and strong net negative charge of the intrabodies at pH 6.6, but not pH 7.4, are critical parameters for stability. To test this hypothesis, we constructed 15 additional s3Flag-scFv-HA proteins derived from scFvs (Supplementary Fig. 2) and quantified their aggregation rate in HeLa cells using an immunocytochemical analysis (Supplementary Fig. 3). We then performed a statistical correlation analysis of the net negative charge and aggregation rate of 16 s3Flag-scFv-HA proteins, including s3Flag-scFv-A36-HA, and scFv-GFPA36 in HeLa cells (a total 17 scFv proteins were tested). We found a statistically significant negative correlation between aggregation and the net negative charge at pH 6.6, but not at pH 7.4 (Fig. 2a); we also found a statistically significant positive correlation between their pI value and aggregation rate (Fig. 2b). These results indicate that the low pI and strong net negative charge of the intrabodies at pH 6.6, but not at pH 7.4, are critical parameters for the stability of cytoplasmic antibodies.

Additionally, the success rate of the stable expression of s3Flag-scFv-HA proteins (16 proteins) was found to be ~82%; 2 constructs showed intense cytoplasmic aggregates (4.9% and 7.8% aggregation) and 14 constructs had fewer aggregates (<2%). Cytoplasmic intrabodies are frequently aggregated and poly-ubiquitinated^21^; thus, these aggregates can be stained with anti-ubiquitin antibodies. We found that scFv-GFP36 aggregates are co-stained with anti-poly-ubiquitin antibodies (Supplementary Fig. 4, arrows). However, no such staining was observed in s3Flag-A36-HA-expressing HeLa cells (Supplementary Fig. 4, arrowheads). These ultra-stable cytoplasmic intrabodies fused with these tags and exhibiting a low pI and a high net negative charge at pH 6.6 are referred to as STANDs.

**Fig. 3:**
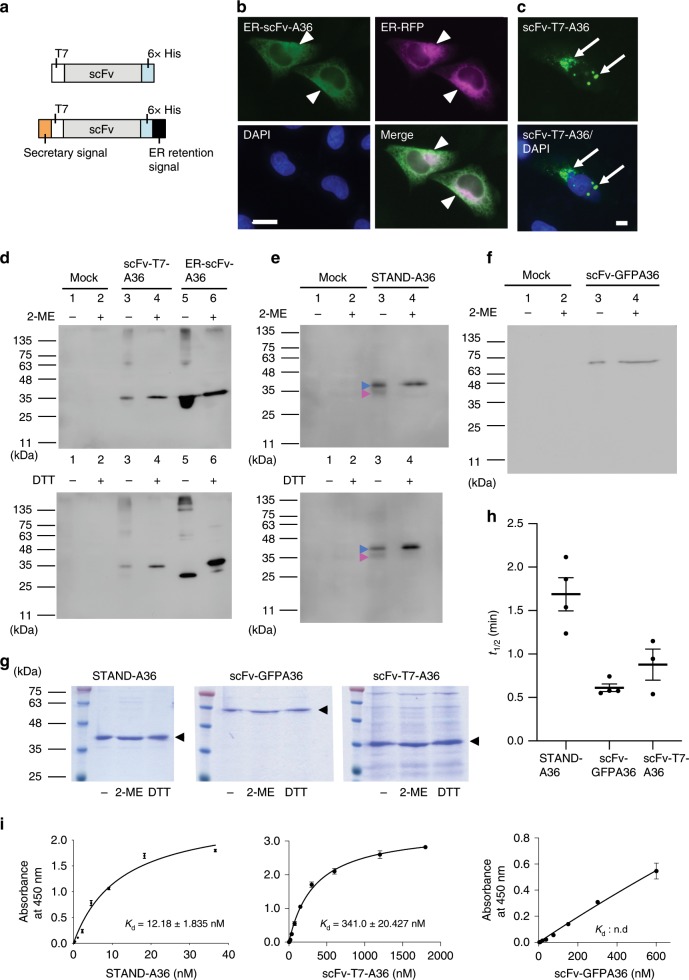
Increased stability and folding rate of STAND-A36. **a** Schematic structure of scFvs used in intra-disulfide bond formation analysis (upper: scFv-T7-A36, lower: endoplasmic reticulum (ER)-targeted scFv-A36 (ER-scFv-A36). **b** Co-localisation of ER-scFv-A36 and ER-RFP in HeLa cells. Arrowheads indicate ER-scFv-A36 in the ER. **c** Expression of scFv-T7-A36 leads to the formation of cytoplasmic aggregates (arrows). Scale bar, 10 µm. **d**–**f** Western blot analysis of the cell lysate of HeLa cells transfected with vectors expressing scFv-T7-A36 **d**, ER-scFv-A36 **d**, STAND-A36 **e**, and scFv-GFPA36 **f** under reduced (6% 2-ME or 10 mM DTT) or non-reduced (2-ME, DTT-free) conditions. Differences in antibody migration distances in SDS-PAGE in reduced and non-reduced conditions were observed only in ER-scFv-A36. **g** SDS–PAGE analysis of the purified STAND-A36 (left), scFv-GFPA36 (middle), and scFv-T7-A36 (right) from *E. coli* cells under reduced (6% 2-ME or 10 mM DTT) or non-reduced (2-ME, DTT-free) conditions. No differences in migration distance were observed in any of the scFvs (arrowheads). **h** Analysis of thermal stability of purified STAND-A36, scFv-GFPA36, and scFv-T7-A36 using a fluorescence dye, PSA. The half-lives (*t*_1/2_) of the cytoplasmic antibodies at 80 °C were examined (*n* = 3–4 independent samples). Statistical analysis: two-tailed one-way analysis of variance (*t*_1/2,_ *F* (2, 8) = 15.1284, *P* *=* 0.001912, Tukey’s multiple comparison test, STAND-A36 vs. scFv-GFPA36: *P* = 0.001816, STAND-A36 vs. scFv-T7-A36: *P* = 0.01465, STAND-A36 vs. scFv-T7-A36: *P* = 0.47826). **i** Binding affinity of purified STAND-A36, scFv-GFPA36, and scFv-T7-A36 to Syt I-C2A (*n* = 3 independent samples per group). Scale bars, 10 μm. Error bars represent standard error of the mean. Source data are provided as a Source Data file.

We further investigated whether the stability and folding of STAND proteins are improved compared to those of other cytoplasmic scFv formats. The intra-disulfide bond formation in the Fv of heavy and light chains plays a major role in protein folding^22^. To investigate the correct formation of intra-disulfide bonds in s3Flag-scFv-A36-HA (termed STAND-A36), we constructed an additional 2 scFvs, scFv-A36 fused with T7- and His-tags (termed scFv-T7-A36, Fig. 3a, upper) as a standard and an endoplasmic reticulum (ER)-targeted scFv-A36 (termed ER-scFv-36) as a positive control (Fig. 3a, lower). In HeLa cells, ER-scFv-A36 was colocalised with ER-RFP, a red fluorescent marker for the ER (Fig. 3b, arrowheads), while scFv-T7-A36, which has a net charge of + 0.6 at pH 6.6, was highly aggregated in the cytoplasm (34.76 ± 3.02%; % cells with aggregates) (Fig. 3c, arrows).

We performed a Western blot analysis with cell lysate from HeLa cells transfected with scFv genes and compared the migration distances of scFv-T7-A36, ER-scFv-A36, STAND-A36, and scFv-GFPA36 in sodium dodecyl sulfate-polyacrylamide gel electrophoresis (SDS–PAGE) under reducing or non-reducing conditions (Fig. 3d–f). The migration distance of ER-scFv-A36 differed in reducing and non-reducing conditions (Fig. 3d), whereas the migration distances of scFv-T7-A36 and scFv-GFPA36 did not (Fig. 3d, f). This indicates that ER-scFv-T7-A36 forms intra-disulfide bonds, but scFv-T7-A36 and scFv-GFPA36 do not. Although the migration distance of a small portion of STAND-A36 (Fig. 3e, magenta arrowheads) differed in the reducing and non-reducing conditions, the migration distance of a large portion of STAND-A36 was the same in both conditions (Fig. 3e, blue arrowheads). This suggests that the majority of STAND-A36 do not form disulfide bonds.

Previous studies reported that several scFvs can fold correctly without intra-disulfide bonds and are stable in the cytoplasm^22^. To investigate whether the STAND method can increase the stability or folding efficiency of cytoplasmic scFvs, STAND-A36, scFv-T7-A36, and scFv-GFPA36 were purified under native conditions of the cytoplasm of *Escherichia coli* (*E. coli*) cells. We found that the migration distances of these cytoplasmic scFvs were the same in both reducing and non-reducing conditions (Fig. 3g), indicating these cytoplasmic scFvs do not form intra-disulfide bonds. We then compared the thermal stability of purified STAND-A36, scFv-T7-A36, and scFv-GFPA36 using a fluorescent dye (termed PSA), that can monitor protein unfolding during heating and has been used for stability assays in previous studies (see Methods)^23^. The hydrophobic region of unfolded, denatured proteins is more exposed on their surfaces than that of native ones. As PSA binds to the hydrophobic surfaces of the proteins and generates fluorescence, the unfolded protein has a higher fluorescence intensity. By incubating the native protein at a raised temperature, a thermal unfolding curve can be observed, and the protein’s half-life (*t*_1/2_) can be calculated and used to evaluate protein stability^23^. The half-life of STAND-A36 (*t*_1/2_ = 1.68 min) at 80 °C was significantly longer than those of scFv-GFPA36 (*t*_1/2_ = 0.612 min) and scFv-T7-A36 (*t*_1/2_ = 0.877 min) (Fig. 3h), indicating that STAND-A36 is more stable than other intrabody formats. An enzyme-linked immunosorbent assay (ELISA) revealed that the binding affinities of scFv-GFPA36 and scFv-T7-A36 to glutathione S-transferase (GST)-Syt I-C2A were significantly lower than that of STAND-A36 (Fig. 3i; *K*_d_ values: STAND-A36, 12.18 nM; scFv-T7-A36, 341 nM; scFv-GFPA36: undetermined, as the saturation curve was not obtained), reflecting a higher rate of correct folding in STAND-A36 than in other formats. Taken together, this shows that fusing s3Flag and HA tags to scFv-A36 can increase its stability and folding efficiency.

### STAND proteins are stably expressed in the cytoplasm in vivo

**Fig. 4:**
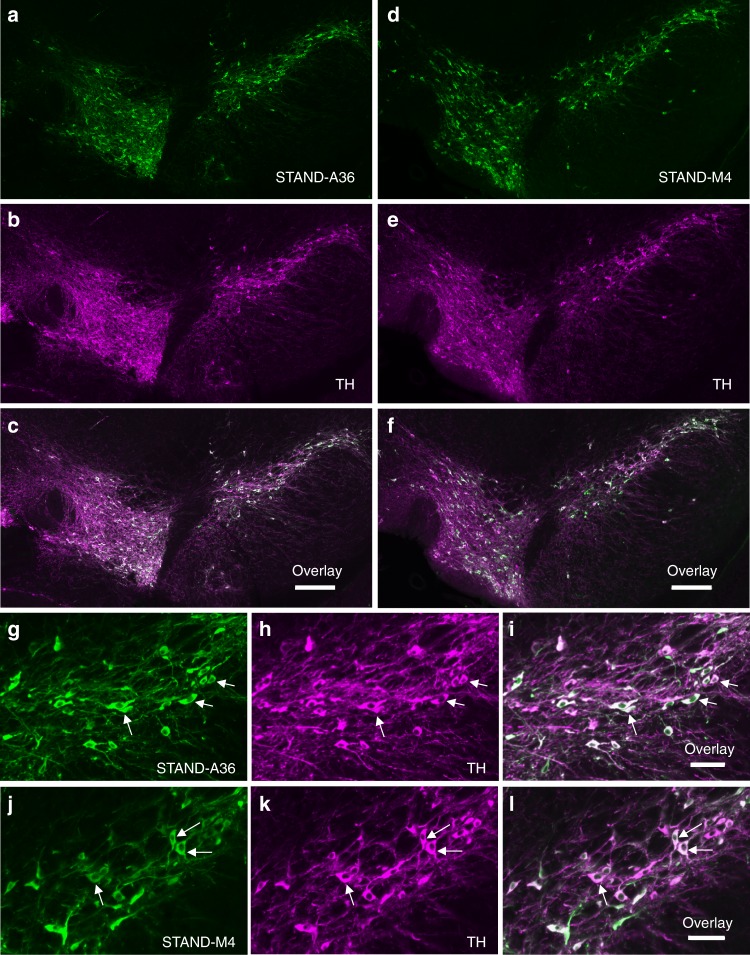
Stable expression of STAND proteins in the SNc. **a**–**l** Immunohistochemical analysis of the SNc 33 days after injection with AAV9/3-STAND-A36 (**a**–**c**, **g**–**i**) or AAV9/3-STAND-M4 (**d**–**f**, **j**–**l**) **a**, **d**, **g**, **j** Anti-Flag antibody to detect scFv protein; **b**, **e**, **h**, **k** anti-tyrosine hydroxylase [TH] antibody used to detect dopaminergic neurons; **c**, **f**, **i**, **l** merged image of **a** and **b**, **d** and **e**, **g** and **h**, or **j** and **k**. **g**–**l** Magnified images of the SNc region in **a**–**c** or **d**–**f**. STAND proteins were stably expressed in the cytoplasm of dopaminergic neurons (arrows). Scale bars, 200 μm **c**, **f** and 50 μm **i**, **l**.

**Fig. 5:**
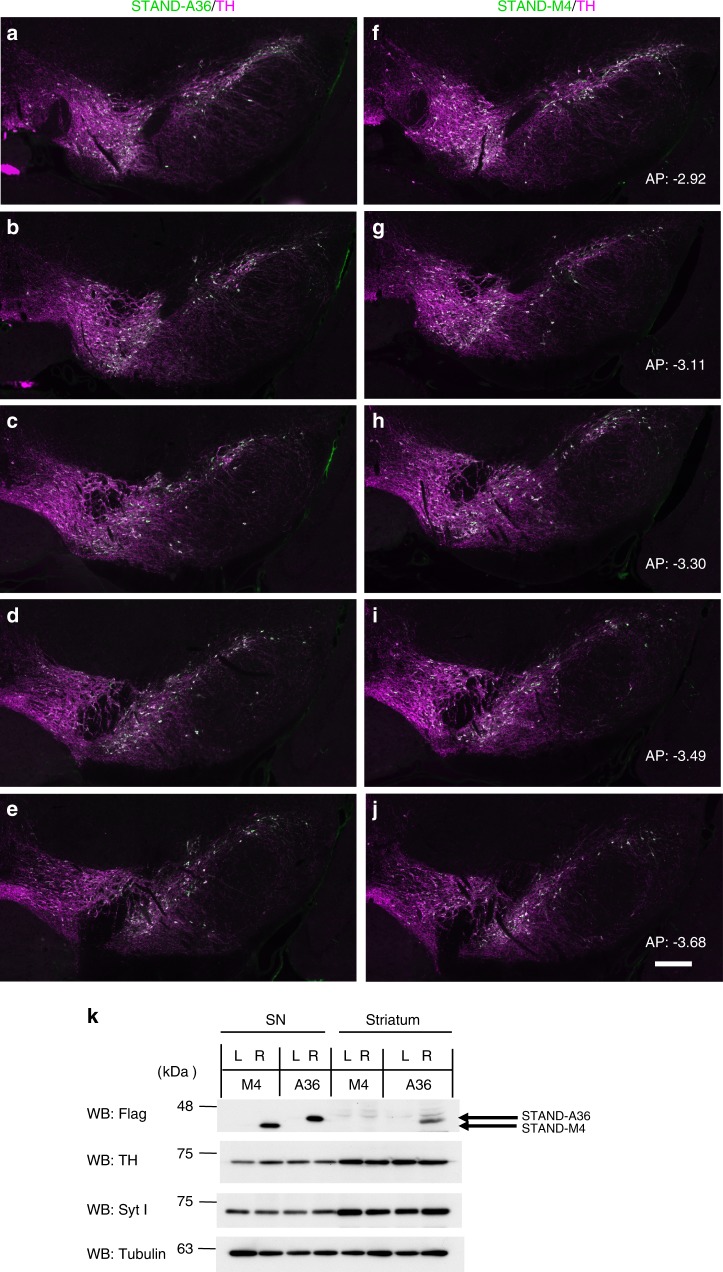
Stable expression of STAND proteins in a broad region of the SNc. Brain sections (AP coordinates relative to bregma in mm: −2.92, −3.11, −3.3, −3.49, and −3.68) 33 days after injection with AAV9/3-STAND-A36 (**a**–**e**) or AAV9/3-STAND-M4 (**f**–**j**). STAND proteins (green) were expressed in tyrosine-hydroxylase (TH)-positive (magenta) dopaminergic neurons (white, in merged images). **k** Immunoblot analysis of total homogenates of the striatum and SNc from the left (L) and right (R) hemispheres of the mouse brain 33 days after injection with AAV-vectors; anti-Flag (STAND proteins), anti-TH, anti-Syt I, and anti-tubulin antibodies. Scale bar, 200 μm.

We constructed AAV9/3 vectors that express STAND proteins under the control of rat synapsin I gene promoter (SynIp). AAV9/3-SynIp-DIO-STAND-A36 (or -STAND-M4; see Methods) was injected into the substantia nigra pars compacta (SNc) of the right midbrain of DAT-Cre heterozygote mice. Immunohistochemical analysis revealed that STAND-A36 and STAND-M4 were stably expressed in the cytoplasm of dopaminergic neurons of the SNc and the ventral tegmental area of the right midbrain hemisphere 33 days after injection (Fig. 4a–l). This expression pattern was observed in dopamine (DA) neurons through the SNc and ventral tegmental area (~768 μm; Fig. 5a–j). We also confirmed the expression of both STAND-A36 and STAND-M4 in the SNc using Western blotting (Fig. 5k, upper panel). The stable expression of both STAND-A36 and STAND-M4 was observed 6 months later without any evidence of neuronal damage (Supplementary Fig. 5a–l, magnified image of 5a–f, arrows), unlike the intracellular aggregates of scFv-GFPA36 detected 30 days after an AAV injection (Supplementary Fig. 6a, arrows). These results were consistent with the propensity of intrabodies in the cytoplasm of HeLa cells (Fig. 1).

Additionally, we found that the expression level of tyrosine hydroxylase in the SNc of a right hemisphere injected with scFv-GFPA36-expressing AAV vector was significantly reduced compared with that in the SNc of a left hemisphere (Supplementary Fig. 6b, upper panel, 6c), suggesting that cytoplasmic scFv aggregates induce DA neuron damage. Nevertheless, no such change was observed in a right hemisphere 6 months after an injection of STAND-A36-expressing AAV vector (Supplementary Fig. 6b, lower panel, 6c).

### Functional performance of STAND-A36 in vitro and in vivo

**Fig. 6:**
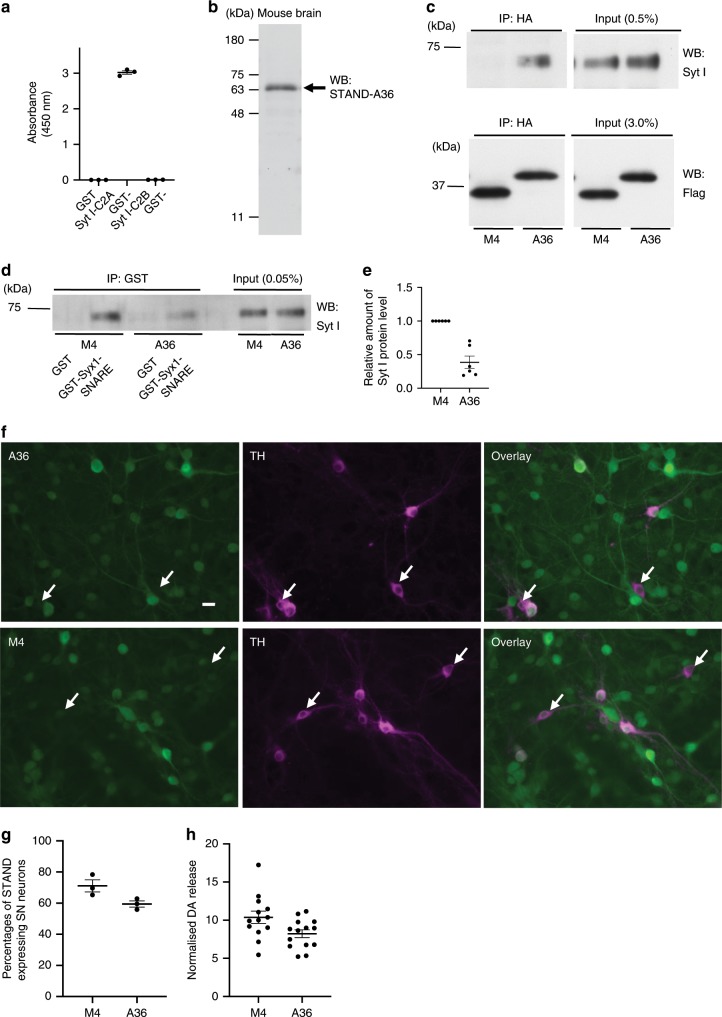
Inhibition of the function of Syt I by STAND-A36 expression in vitro. **a** Binding of purified STAND-A36 to indicated GST-fused proteins, as determined by an enzyme-linked immunosorbent assay (*n* = 3 independent samples per group). Statistical analysis: two-tailed one-way ANOVA: absorbance, *F* (2, 6) = 3497, *P* < 0.00001; Tukey’s multiple comparison test: GST-C2A vs. GST-C2B: *P* < 0.00001, GST-C2A vs. GST: *P* < 0.00001, GST vs. GST-C2B: *P* = 0.98987). **b** Western blot analysis of total mouse brain homogenates using purified STAND-A36 as a primary antibody. **c** Intracellular interaction of STAND-A36, but not of STAND-M4, with Syt I expressed in 293 T cells co-transfected with Syt I-expressing vector and indicated vectors. Immunoprecipitates obtained using anti-HA antibody were analysed using western blotting with an anti-Flag antibody to detect scFv and anti-Syt I antibodies. **d** Decreased interaction of Syt I with the Syx1B-SNARE domain by the expression of STAND-A36. The cell lysate described in **c** was incubated with purified GST or GST-Syx1-SNARE, then subjected to immunoprecipitation with the anti-GST antibody. Immunoprecipitates were analysed using western blotting with an anti-Syt I antibody. **e** Quantification of the relative amount of Syt I protein co-immunoprecipitated with GST-Syx1-SNARE described in **d** (*n* = 8 independent experiments per group). Statistical analysis: Mann-Whitney *U* test, *P* = 0.0021645. **f**–**h** STAND-A36 expression in cultured dopamine neurons inhibited high potassium-induced dopamine release. **f** Fluorescent images of dopaminergic neurons (DIV 14) expressing STAND-A36 (upper panels) and STAND-M4 (lower panels) (Left: anti-HA antibody to detect STAND proteins; middle: anti-TH antibody to detect dopaminergic neurons, right: merged images of left and middle panels). Arrows show dopaminergic neurons, which did not express STAND proteins. Scale bar, 20 μm. **g** Transfection efficiency of STAND proteins in dopaminergic neurons. **h** Measurement for high potassium-induced dopamine release normalised with a low potassium-induced one in cultured dopamine neurons. Statistical analysis: two-tailed *t*-test, *P* = 0.0311. Error bars represent standard error of the mean. Source data are provided as a Source Data file.

Next, we investigated the effects of STAND-A36 on the function of Syt I. ELISA results revealed that purified STAND-A36 specifically recognised the C2A domain of Syt I, but not the C2B domain (Fig. 6a); also, putative mouse brain Syt I/II was shown (Fig. 6b, arrow). These results indicate that STAND-A36 is a highly specific intrabody with high target affinity (Fig. 3, 12.18 nM). We then investigated whether intracellular STAND-A36 can bind to Syt I in the cytoplasm of 293 T cells. Syt I co-immunoprecipitated with STAND-A36, but not with STAND-M4 (Fig. 6c).

The C2A domain of Syt I interacts with the soluble *N*-ethylmaleimide-sensitive factor attachment protein receptor (SNARE) domain of Syntaxin1, which is important for neurotransmitter release^24^. To investigate whether STAND-A36 inhibits this interaction, we subjected the cell lysate described in Fig. 6c to GST pulldown and used Western blotting to analyse the proteins that precipitated with purified GST-syntaxin1-SNARE (GST-Syx1-SNARE). Co-precipitation of Syt I and GST-Syx1-SNARE was significantly decreased to ~46.5% in cells expressing STAND-A36 compared to those expressing STAND-M4 (Fig. 6d, e), indicating that STAND-A36 interferes with the interaction between Syt I and Syntaxin I by binding to the C2A domain of Syt I.

A previous study demonstrated that Syt I plays a major role in axonal DA release in cultured DA neurons and contributes to around 40% of all DA release, while Syt VII is a second isoform contributing to around 20% of all DA release^25^. To examine the effect of STAND-A36 expression on DA release, a DA neuron culture was infected with an AAV vector expressing STAND-A36, and high potassium (60 mM)-induced release was measured 7 days after AAV infection. The transduction efficiencies of STAND-A36 and STAND-M4 into DA neurons were ~59% and 71%, respectively (Fig. 6f, g). The low transduction efficiency might be due to the general property of AAV vectors, whose transduction efficiency in vitro is different from that in vivo^26^. The high potassium-induced DA release in AAV1-STAND-A36-infected cultures was significantly reduced to 79.3% compared to that in STAND-M4 (Fig. 6h), indicating that STAND-A36 inhibits Syt-I-dependent DA release.

**Fig. 7:**
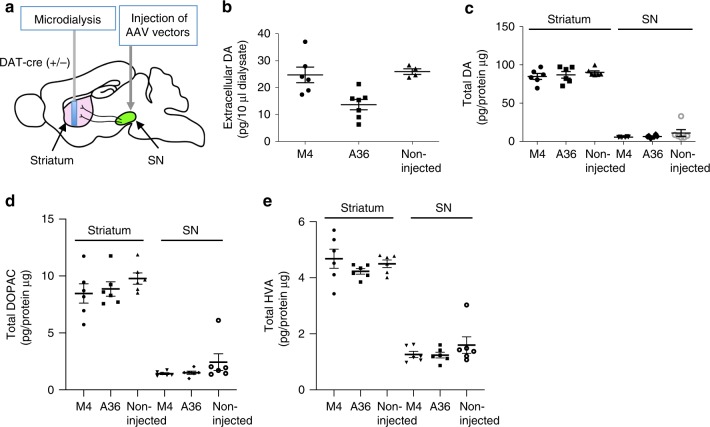
STAND-A36 inhibits striatal dopamine release. **a**–**e** Intracellular expression of STAND-A36 decreases basal dopamine release in the striatum in vivo. **a** Microdialysis was performed in the striatum of the right cerebral hemisphere of mice 33 days after AAV injection into the SNc. **b** Average basal extracellular dopamine levels in the striatum of awake mice were measured every 10 min over a 60-min period using microdialysis (M4: *n* = 6 mice/group, A36: *n* = 7 mice/group, non-injected: *n* = 4 mice/group). Statistical analysis: two-tailed one-way ANOVA, *F* (2, 14) = 9.2457, *P* = 0.002757, A36 vs. M4, *P* = 0.0071; A36 vs. non-injected, *P* = 0.0079; M4 vs. non-injected, *P* = 0.9352. **c**–**e** Total dopamine, dopamine metabolite (DOPAC and HVA) levels in the striatum and SNc of the right cerebral hemisphere of mice with or without an injection of AAV vectors (*n* = 6 mice/group). Statistical analysis: two-tailed one-way ANOVA, **c** Dopamine in striatum, *F* (2, 15) = 0.5833, *P* = 0.5702; dopamine in SN, *F* (2, 15) = 1.2156, *P* = 0.3241; **d** DOPAC in striatum, *F* (2, 15) = 0.99535, *P* = 0.392734; DOPAC in SN, *F* (2, 15) = 1.633946, *P* = 0.228045; **e** HVA in striatum, *F* (2, 15) = 1.0771461, *P* = 0.3655; HVA in SN, *F* (2, 15) = 1.1143889, *P* = 0.353814. Error bars represent standard error of the mean. Source data are provided as a Source Data file.

We then investigated the in vivo performance of STAND-A36 in the DA system of mouse striatum. Basal DA release in the striatum of rats or mice is dependent on extracellular Ca^2+^and nerve-action potential^27,28^, suggesting that it involves Syt I. To assess the inhibitory activity of STAND-A36 against endogenous Syt I in dopaminergic SNc neurons in vivo, we measured the basal DA release in the striatum of STAND-A36-expressing, STAND-M4-expressing, and non-AAV9/3-injected (control) mice at 33 days using microdialysis (Fig. 7a). We found that extracellular DA was significantly decreased in STAND-A36-expressing mice compared to that in STAND-M4-expressing and control mice (Fig. 7b). In contrast, the total amount of DA and DA metabolites (3,4-dihydroxyphenylacetic acid [DOPAC] and homovanillic acid [HVA]) in the striatum and substantia nigra did not differ among the 3 groups (Fig. 7c–e). Together, these results indicate that STAND-A36 inhibits striatal DA release in vivo.

**Fig. 8:**
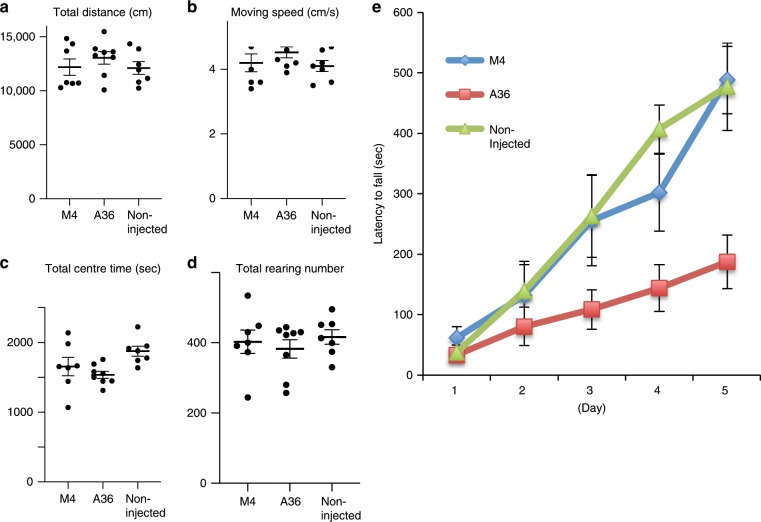
STAND-A36 impairs motor-skill learning. **a**–**d** Open-field test. Total distance travelled **a**, moving speed **b**, total time spent in the centre of the field **c**, and total number of rearings **d** are shown. **e** Rotarod test. Latency to fall is shown. Statistical analysis in **a**–**d**; two-tailed one-way ANOVA: Total distance, *F* (2, 19) = 0.6885, *P* *=* 0.51441; moving speed, *F* (2, 19) = 1.19321, *P* *=* 0.32476; total number of rearings, *F* (2, 19) = 0.40367, *P* *=* 0.67345; total centre time, *F* (2, 19) = 3.79839, *P* *=* 0.04095, Tukey’s multiple comparison test, A36 vs. non-injected, *P* = 0.033782; A36 vs. M4, *P* = 0.612174; M4 vs. non-injected, *P* = 0.223909. Statistical analysis in **e**; two-tailed two-way ANOVA: interaction, *F* (8, 76) = 2.890, *P* *=* 0.0072677; gene, *F* (2, 19) = 6.208, *P* *=* 0.00842; time, *F* (2.771, 52.66), *P* < 0.00001, Tukey’ multiple comparison test, Day 5, A36 vs. M4, *P* *=* 0.0053325; A36 vs. non-injected, *P* *=* 0.0247076; M4 vs. non-injected, *P* *=* 0.9933278, M4, Day 1 vs. Day 5, *P* *=* 0.002306; A36, Day 1 vs. Day 5, *P* *=* 0.0885538; non-injected, Day 1 vs. Day 5, *P* *=* 0.007349. Error bars represent standard error of the mean. Source data are provided as a Source Data file.

To further investigate the functional significance of STAND-A36, we subjected mice to behavioural testing. STAND-A36- or STAND-M4-expressing AAV vectors were bilaterally injected into the substantia nigra of DAT-Cre mice (Fig. 8a–e). An immunohistochemical analysis of the brain 4 weeks after injection confirmed their bilateral expression in the dopaminergic neurons of the SNc (Supplementary Fig. 7). In an open-field test, except for the amount of time spent in the centre of the open field (STAND-A36 vs. non-injected, *P* = 0.04095), no statistically significant differences were noted among the groups in terms of the total distance covered, movement speed, and rearing frequency (Fig. 8a–d).

Next, we evaluated motor skills with a rotarod test: we analysed the latency to fall before (Day 1) and after (Day 5) training using a two-way repeated measures ANOVA (Fig. 8e). Motor-skill learning was impaired in STAND-A36-expressing mice (Fig. 8e), whereas non-injected and STAND-M4-expressing mice showed improved motor skills 5 days after training (Fig. 8e). Moreover, STAND-M4-expressing and non-injected mice stayed on the rod longer than STAND-A36-expressing mice at Day 5 (Fig. 8e). Latency to fall at Day 1 did not differ significantly among groups (Supplementary Table 1).

### The generalisation of the STAND method

**Fig. 9:**
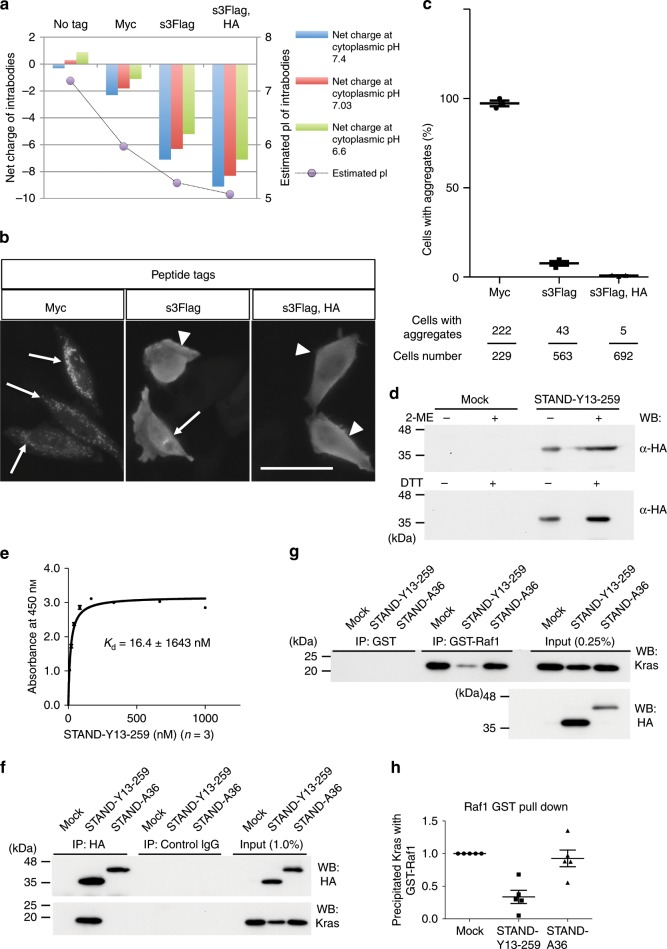
Convertion of anti-Kras scFv-Y13-259 to high-affinity STAND. **a** In-silico analysis of net charge (left axis) of Y13-259 fused with indicated peptide tags at cytoplasmic pH 6.6 (green), 7.03 (red), and 7.4 (blue), and pI (grey circles, right axis). **b** Intracellular stability comparison of scFv-Y13-259 fused with different peptide tags expressed in HeLa cells using immunocytochemistry; anti-Flag or anti-myc was used to detect scFv proteins. Arrows indicate cytoplasmic scFv aggregates. Arrowheads indicate stable expression of scFv proteins. Scale bar, 50 μm. **c** Percentage of scFv-expressing cells with aggregates in **b** (upper panel). Bottom panel shows total-cell and aggregated-cell numbers from 3 independent experiments. Statistical analysis: two-tailed one-way analysis of variance (ANOVA), *F* (2, 6) = 2110.0416, *P* < 0.00001, Tukey’s multiple comparison test, s3Flag-Y13-259-HA vs. s3Flag-Y13-259, *P* *=* 0.0141; s3Flag-Y13-259 vs. Myc-Y13-259, *P* < 0.00001; s3Flag-Y13-259-HA vs. Myc-Y13-259, *P* < 0.00001. **d** Western blot of cell lysate of HeLa cells transfected with vectors expressing STAND-Y13-259 under reduced (upper panel: 6% 2-ME, lower panel: 10 mM DTT) or non-reduced (2-ME, DTT-free) conditions. **e** Binding affinity of purified STAND-Y13-259 with GST-Kras measured by ELISA (*n* = 3 independent samples). **f** Immunoprecipitation analysis using 293 T cells co-transfected with Kras-expressing vector and indicated vectors; western blotting with an anti-HA antibody to detect scFv and anti-Kras antibodies. **g** Decreased interaction of Kras with the Ras-binding domain (RBD) of Raf1 by the expression of STAND-Y13-259; 293 T cells were transfected with Kras- and indicated STAND-expressing vectors. One day after transfection, the cell lysate was incubated with purified GST or GST-Raf1-RBD, then subjected to immunoprecipitation with anti-GST antibody. Immunoprecipitates were analysed using western blotting with an anti-Kras antibody. **h** Quantification of the relative amount of Kras protein co-immunoprecipitated with GST-Raf1-RBD (*n* *=* 5 independent samples). Precipitated Kras was normalised with the total Kras protein. Statistical analysis: two-tailed one-way ANOVA, *F* (2, 12) = 14.9438, *P* *=* 0.0005527, Tukey’s multiple comparison test, Mock vs. STAND-Y13-259, *P* *=* 0.009; Mock vs. STAND-A36, *P* *=* 0.8472; STAND-Y13-259 vs. STAND-A36, *P* *=* 0.0022. Error bars represent standard error of the mean. Source data are provided as a Source Data file.

To assess the generalisability of our methodology, we used the STAND method to intervene in the molecular oncogenic pathway. Previously, a neutralising rat monoclonal antibody, Y13-259 whole IgG, was developed^29–31^ against Ras small GTPases (Kras, Hras, and Nras), which have an oncogenic function^32^. However, cytoplasmic myc-tag-fused scFv-Y13-259 (termed Myc-Y13-259) formed insoluble aggregates in mammalian cells^6^ and failed to show a direct interaction with intracellular endogenous or exogenous Kras in mammalian cells via co-immunoprecipitation^6^. Thus, we attempted to convert Myc-Y13-259 into a STAND. As expected, an in-silico analysis revealed that fusion of s3Flag alone or of both s3Flag and HA tags to scFv-Y13-259 strongly decreased the pI to 5.29 or 5.08, respectively, and increased the net negative charge to –5.2 or –7.1 at pH 6.6, respectively, whereas fusion of the myc tag did not (Fig. 9a). Immunocytochemical analysis revealed that Myc-Y13-259 was highly aggregated in the cytoplasm of HeLa cells (97.21 ± 1.28%; Fig. 9b, arrows, c), as described previously^6^. Fusion of the s3Flag alone reduced the percentage of aggregation to ~7.64 ± 1.03% (Fig. 9b, c); however, its effect was low, similar to that in the case of scFv-A36 or scFv-M4 fused with the s3Flag alone (Fig. 1). This suggests that the stability needs to be improved. Fusion of s3Flag and HA tags to scFv-Y13-259 (termed s3Flag-Y13-259-HA) decreased the percentage of aggregation to 0.75 ± 0.16%, which was significant compared to that of the other constructs (Fig. 9b, arrowheads, c). Therefore, s3Flag-Y13-259-HA was termed STAND-Y13-259. In addition to STAND-A36 and the other cytoplasmic scFvs shown in Fig. 3, STAND-Y13-259 does not form intra-disulfide bonds, as there is no difference in the migration distance in the SDS–PAGE results under reducing and non-reducing conditions (Fig. 9d).

STAND-Y13-259 was expressed in the cytoplasm of *E. coli* and could be purified under the native condition. We could measure the half-life of STAND-Y13-259 using PSA (*T*_1/2_ = 1.11 ± 0.37 min, *n* = 4); however, we could not compare the stability of STAND-Y13-259 and Myc-Y13-259 because cytoplasmic Myc-Y13-259 formed aggregates in *E. coli*, and could not be purified in adequate quantities for biochemical studies (Supplementary Fig. 8). ELISAs performed showed that the *K*_d_ of STAND-Y13-259/purified GST-fused Kras was ~16.4 nM (Fig. 9e), suggesting the high rate of its folding in the cytoplasm and potent inhibitory effects of STAND-Y13-259 on the Kras-mediated oncogenic signalling pathway. One of the Kras signalling pathways for cancer growth is mediated by its downstream molecule, Raf1, via the interaction of the Ras-binding domain (RBD) of Raf1 (Raf1-RBD) and activated Kras; Y13-259 whole IgG interferes with this interaction^33^. Next, we investigated the effect of STAND-Y13-259 on this interaction using 293 T cells. Kras- and STAND-Y13-259-expressing vectors were co-transfected into 293 T cells, and the cell lysate was subjected to co-immunoprecipitation and GST-pulldown analysis (Fig. 9f–h). Co-immunoprecipitation revealed that intracellular STAND-Y13-259 interacts with Kras, but STAND-A36 does not (Fig. 9f). The proteins that precipitated with purified GST-fused Raf1-RBD (GST-Raf1-RBD) were analysed using Western blotting (Fig. 9g). Co-precipitation of Kras with GST-Raf1-RBD was significantly decreased in cells expressing STAND-Y13-259 when compared to those expressing STAND-A36 or the mock vector control (Fig. 9g, h), indicating that intracellular STAND-Y13-259 interferes with the interaction of activated Kras and Raf1 by binding to Kras.

**Fig. 10:**
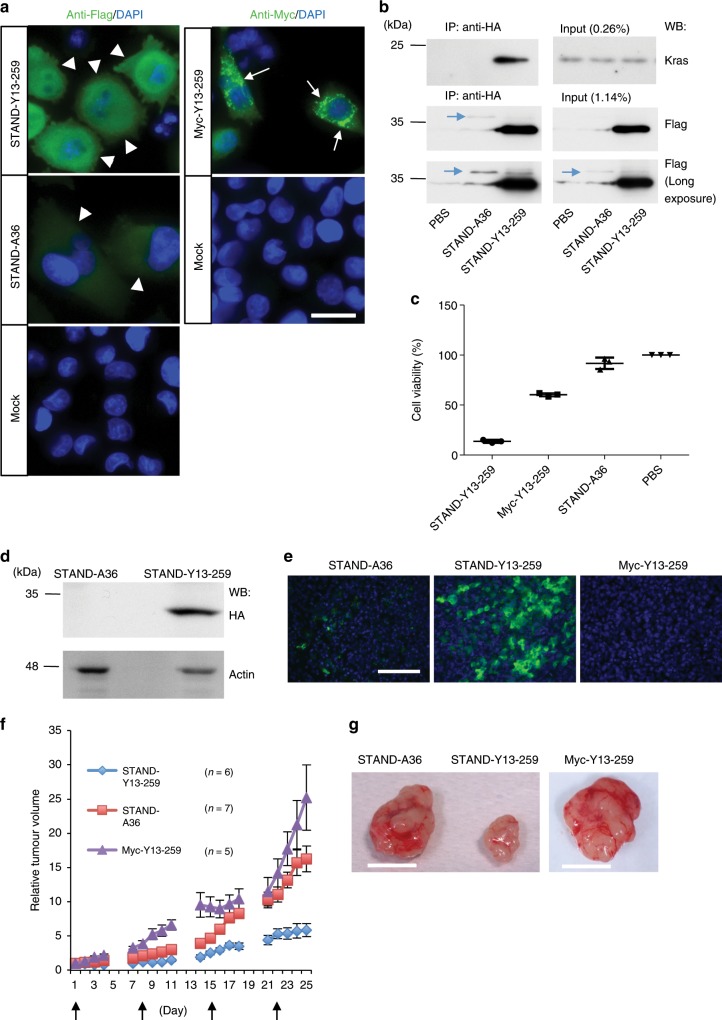
Tumour growth inhibition by STAND-Y13-259 in vivo. **a** Immunocytochemical analysis of MIA PaCa-2 cells 4 days after infection with Lenti-STAND-Y13-259, Lenti-Myc-Y13-259, and Lenti-STAND-A36 (green; anti-Flag to detect scFv; blue, Hoechst staining to detect nuclei). Arrowheads indicate STAND-Y13-259 and STAND-A36 stably expressed in the cytoplasm. Arrows indicate Myc-Y13-259 aggregates. **b** Intracellular interaction of endogenous Kras and STAND-Y13-259 in MIA PaCa-2 cells infected with lentiviral vectors. Immunoprecipitates with anti-HA antibody were analysed using western blotting (WB) with indicated antibodies. Expression of STAND-A36 was weak compared to that of STAND-Y13-259 (arrows in middle and bottom panels). **c** Viability of MIA PaCa-2 cells infected with indicated lentiviral vectors, as determined using an MTS assay after 4 days (*n* = 3 independent samples). Statistical analysis: two-tailed one-way analysis of variance, *F* (3, 8) = 490.4608, *P* < 0.00001, Tukey’s multiple comparison test, STAND-Y13-259 vs. Myc-Y13-25, *P* < 0.00001; STAND-Y13-259 vs. STAND-A36, *P* < 0.00001; STAND-Y13-259 vs. phosphate-buffered saline (PBS), *P* < 0.00001; Myc-Y13-259 vs. PBS, *P* < 0.00001; STAND-A36 vs. PBS, *P* *=* 0.0641. **d** Western blotting using a tumour of a subcutaneous heterotopic xenograft model of pancreatic cancer. Pre-established tumours were injected once weekly for 4 weeks at 2 sites with lentiviral vectors. Western blot analysis of STAND-Y13-259 (upper panel) and actin (lower panel) expression in tumours dissected from mice 3 days after the fourth lentiviral vector injection. **e** Immunohistochemistry of STAND-Y13-259 (upper panel), STAND-A36 (middle), and Myc-Y13-259 (lower panel) in the tumour described in **d**. **f** Tumour growth as described in **d**. Arrows indicate the day of lentivirus injection. Statistical analysis: Kruskal–Wallis test, *P* *=* 0.0005310, Dann’s post-hoc multiple comparisons test (STAND-Y13-259: *n* *=* 6, Myc-Y13-259: *n* *=* 5, STAND-A36: *n* = 7, STAND-Y13-259 vs. Myc-Y13-259, *P* *=* 0.0052; Myc-Y13-259 vs. STAND-A36, *P* > 0.9999; STAND-Y13-259 vs. STAND-A36, *P* *=* 0.0269). **g** Representative digital photographs of tumour xenografts dissected from mice 24 days after the first intratumoural injection with indicated lentiviral vectors. Scale bars, 25 μm **a**, 100 μm **e** and 10 mm **g**. Error bars represent standard error of the mean. Source data are provided as a Source Data file.

To further validate the functional significance of STAND-Y13-259 in Kras-mediated signalling, we constructed lentiviral vectors to express the intrabodies in MIA PaCa-2 human pancreatic cancer cells that are homozygous for an oncogenic Kras mutation (G12C)^34^. STAND-Y13-259 and STAND-A36 were stably expressed in the cytoplasm of MIA PaCa-2 cells 4 days after infection (Fig. 10a, upper left panel and middle left panel, arrowheads); in contrast, Myc-Y13-259 formed aggregates (Fig. 10a, upper right panel, arrows). Endogenous Kras in MIA PaCa-2 cells co-immunoprecipitated with STAND-Y13-259, but not with STAND-A36 (Fig. 10b). We were unable to investigate the interaction of Myc-Y13-259 with endogenous Kras, since it was not recoverable in the soluble form from the mammalian cells^6^.

Microinjection of Y13-259 whole IgG has been shown to induce cancer-cell death^31^. Therefore, we investigated the effect of cytoplasmic STAND-Y13-259 expression on the proliferation of MIA PaCa-2 cells. We found that, unlike Myc-Y13-259 or STAND-A36, STAND-Y13-259 strongly inhibited cell proliferation (Fig. 10c). Next, we assessed the in vivo performance of STAND-Y13-259 in pre-established subcutaneous MIA PaCa-2 tumours in nude mice. Stable STAND-Y13-259 expression was confirmed using Western blotting and immunohistochemistry 24 days after the first infection (Fig. 10d, e). Weekly injections of STAND-Y13-259-expressing lentiviral vector into pre-established subcutaneous MIA PaCa-2 tumours in nude mice strongly inhibited tumour growth when compared to injections of STAND-A36 or Myc-Y13-259 (Fig. 10f, g).

## Discussion

The STAND method has an advantage over other intrabody formats in converting aggregation-prone scFvs to stable cytoplasmic antibodies; a number of scFvs isolated in last 30 years would require only the fusion of s3Flag and HA peptide tags to be available for in vitro and in vivo applications. Using the STAND method, we created an ultra-stable anti-Kras cytoplasmic intrabody with high affinity from a previously isolated aggregation-prone intrabody without the need for amino-acid substitutions (Figs. 9 and 10). Contrastingly, conventional approaches, including CDR grafting to stable frameworks and/or structure-based framework engineering, can improve the stability and/or folding efficiency of intrabodies only to a limited extent^35^. Moreover, amino-acid residues in CDRs affect not only antigen-binding specificity and affinity, but also protein stability in the cytoplasm^4^. Methods for engineering a CDR that can increase the stability of cytoplasmic intrabodies without altering their affinity and specificity are lacking.

It appears that STAND proteins do not form intra-disulfide bonds in the cytoplasm (Figs. 3 and  9). Nevertheless, they are stable enough to inhibit the target protein function. Worn and Pluckthun reported an intrinsically stable scFv fragment without intra-disulfide bonds^36^. Recently, Gąciarz and Ruddock reported that 2 cytoplasmic scFvs can fold independent of disulfide bonds, and that this folding depends on the framework region and the CDRs^22^. This suggests that correct folding is affected by an unknown mechanism in addition to intra-disulfide bonds and indicates that the strong net negative charge on STANDs even at pH 6.6 may affect this unknown mechanism to stabilise the cytoplasmic antibodies. Further studies are needed to elucidate this mechanism.

Unfortunately, it is generally thought that classical cytoplasmic scFvs or scFvs secreted to the periplasm in *E. coli*, as well as in animal cells, are highly aggregated or poorly expressed; hence, it is often difficult to purify a sufficient amount of protein for biochemical studies (Supplementary Fig. 8)^5,8,37–40^. Therefore, at present, it seems impossible to judge whether the STAND method can systematically create cytoplasmic scFvs with stability similar to parental scFvs with intra-disulfide bonds. Nevertheless, we think that our method can develop and screen very useful cytoplasmic scFvs by comparing the thermal stability and binding affinity. Indeed, STAND proteins were easily purified from soluble fractions of *E. coli*, and their half-lives and binding affinities were determined (Figs. 3 and 9). These binding affinities were not low when compared with the affinity of the reported antibody drugs for practical use (e.g., *K*_d_, Herceptin; 5–7 nM, Rituxan; 8 nM)^41–43^, suggesting that the correct folding rates of these STAND proteins are relatively high. Thus, for successful in vivo applications, it may be important to screen and isolate STAND proteins with a nanomolar range of K_d_ values and the highest half-life values from a STAND library with low pIs and strong net negative charges at pH 6.6 by fusing appropriate peptide tags such as s3Flag and HA.

Notably, oncogenic mutations in Hras, Kras, and Nras, are common in approximately 25% of human cancers, and Kras mutations have been identified in 86% of Ras-driven cancers, including about 98% of pancreatic and 53% of colorectal cancers^44^. However, the flat surface of Ras proteins that precludes the binding of small-molecule inhibitors has hindered the development of effective drugs for cancer treatment, since the affinity of the reported small compounds is in the micromolar range^45–48^ and below the required value for clinical applications^49^. However, purified STAND-Y13-259 showed high affinity for Kras (Fig. 9). Several clinical trials have recently shown that oncolytic viruses such as herpes simplex virus type 1, which can infect and replicate only in oncogenic cancer cells, induce cancer-cell death^50^. Thus, it may be worth exploring whether a combination of STAND-Y13-259 with oncolytic viruses can establish a tumour-specific expression of STAND-Y13-259 and enhance cancer-cell-death activity.

Cytoplasmic expressed scFv-GFPA36 formed aggregates and induced dopaminergic neuron damage in vivo (Supplementary Fig. 6). This is probably because scFv-induced aggregates disrupt the ubiquitin–proteasome system^21^. However, STAND proteins were stably expressed without forming aggregates in the cytoplasm of dopaminergic neurons in vivo 33 days after AAV injection (Figs. 4 and 5), with no apparent differences in the total striatum, SNc DA contents, or the amount of DA released when compared to those of non-injected control mice (Fig. 7). These results indicate that neither AAV injections nor STAND expression in the cytoplasm affected dopaminergic neuron viability or DA release. Furthermore, no STAND aggregation or neuronal damage was noted in the SNc and striatum 6 months after AAV injection (Supplementary Fig. 5 and 6), indicating that STAND was non-toxic and ultra-stable in the mammalian brain. These stability and non-toxicity features of STAND-A36 are essential for analysing target protein function in specific neural circuits.

Syt I functions as a Ca^2+^ sensor for DA release from axonal terminals in cultured DA neurons and PC12 cells; however, whether Syt I acts as a Ca^2+^ sensor in in vivo striatal DA release remains unknown. Thus, our results on STAND-A36 (Figs. 3−8) show the positive role of Syt I, both in striatal DA release and motor-skill learning. However, we could not exclude the possibility that reduced extracellular DA in STAND-A36-expressing mice might be indirectly caused, in part, by changing the reuptake of extracellular striatum DA through the increased activity or expression of DAT or other SNARE proteins^51^.

We found that mice selectively expressing STAND-A36 in DA neurons of the SNc showed normal motor activity but had motor-skill learning deficits (Fig. 8). Since DA release in the striatum of mice expressing STAND-A36 was inhibited by 55.3% compared with that in mice expressing STAND-M4 (Fig. 7), we concluded that striatal DA release is indispensable for motor-skill learning. Mice expressing STAND-A36 in SNc dopaminergic neurons could be used to develop therapeutic cues against Parkinson’s disease and elucidate mechanisms of motor-skill learning.

In contrast, normal motor behaviour is likely not severely sensitive to the decrease in DA release in the striatum (Fig. 8a−d). Previous studies revealed that a change in locomotor function depends on how reduced DA release is induced; depletion of dopaminergic neurons in adult animals by administrating neurotoxins such as 6-hydroxydopamine (6-OHDA) leads to reduced motor activity, but produces less severe deficits in locomotor activity in neonatal rats^52^ and a developmental reduction of striatal DA with dopaminergic-neuron-specific expression of diphtheria toxin^53^ or with dopaminergic-neuron-specific deletion of Ndufs4^54^ produces no difference in locomotor activity. Similarly, recent studies show that the motor symptoms of Parkinson’s disease patients only become evident when the striatum DA contents are decreased to <30−40%^55^. These findings suggest the existence of compensatory or adaptive mechanisms during development or a non-acute depletion of striatal DA.

Also, AAV vector-mediated expression of STAND-A36 might gradually increase over time; therefore, inhibition of Syt I/II by STAND-A36 could be achieved non-acutely, which could lead to an adaptation to maintain basal locomotor activity. It has been suggested that one such adaptation mechanisms is mediated by post-synaptic striatal DA receptor sensitisation because L-DOPA (L-3,4-dihydroxyphenylalanine) administration strongly increased locomotor activity in the aforementioned diphtheria toxin-expressing mice compared with control mice^53^. Thus, it is important to investigate whether striatal DA receptor sensitisation involves an adaptation in STAND-A36-expressing mice for locomotor activity.

## Methods

### Cell culture

HEK293T, HeLa-S3, COS-7, and highly malignant MIA PaCa-2 pancreatic adenocarcinoma cells obtained from RIKEN Bioresource Center Cell Bank (Tsukuba, Japan) were cultured in Dulbecco’s modified Eagle’s medium (DMEM; Wako Pure Chemical Industries, Osaka, Japan; #044-29765) supplemented with 10% fetal bovine serum (FBS; Gibco, Grand Island, NY, USA). Primary dopaminergic neuron cultures were prepared from the ventral mesencephalon of embryonic day 13–14 male and female mouse embryos. Briefly, the ventral mesencephalon was dissociated by treatment with trypsin (0.25% for 20 min at 37 °C; Gibco), followed by trituration with a fire-polished Pasteur pipette in neurobasal medium (Gibco) supplemented with 10% FBS containing deoxyribonuclease I (DNase I). Dissociated cells (6 × 10^4^) were seeded on poly-l-lysine-coated (1 μg/ml) glass coverslips (diameter: 12 mm; Thermo Fisher Scientific, Waltham, MA, USA) in a 24-well plate (Falcon; Corning, NY, USA) and cultured in 500 μl of neurobasal medium supplemented with B27 (Invitrogen, Carlsbad, CA, USA). At 7 days in vitro, cells in each well were infected with 5 μl AAV vector (titre: 1 × 10^11^ vg/ml) to express STAND proteins in a neuron-specific manner by a synapsin promoter and analysed by immunocytochemistry or used for the DA release assay 7 days later.

### In vivo animal studies

All experimental procedures were performed in accordance with the guidelines of the Animal Experiment Committee of RIKEN CBS. Mice were housed on a 12:12-h light/dark cycle, with the dark cycle occurring from 20:00 to 08:00 hours. Dat + /IRES-Cre mice^56^ were purchased from Jackson Laboratory (Bar Harbor, ME, USA). We used AAV vectors expressing scFv genes along with a DAT-Cre mouse line to selectively express antibody genes in dopaminergic neurons of the SNc. Dat + /IRES-Cre mice (male, age: 2 months) injected with viral vectors for microdialysis and behavioural tests were male heterozygote littermates of mated heterozygotes. Balb/c, Balb/c-nu, and wild-type C57B6/J mice were purchased from Charles River Japan (Yokohama, Japan).

### Alignment of scFv proteins with scFv-A36

Multiple alignments of amino-acid sequences of scFv proteins were made using CLUSTALW software v2.1 (http://clustalw.ddbj.nig.ac.jp/index.php?lang = ja)^57^. The accession numbers of the 94 scFv proteins obtained using a BLAST search for scFv-A36 are listed in Supplementary Table 2.

### Antibodies

Antibodies used in the study are listed in Supplementary Table 3.

### Isolation of antigen-reactive phages

In scFv, the heavy- and light-chain variable regions (VH and VL, respectively) of the antibody are fused by a glycine linker encoded by a single gene. The Recombinant Phage Antibody System (GE Healthcare, Little Chalfont, UK) allows large repertoires of scFv to be displayed on the surface of M13 phages. The total RNA was isolated from the spleen of Balb/c mice (female, age: 2 months) immunised with GST-Syt II-C2A^58,59^. The VH and VL were amplified in 2 separate reactions using degenerate primers (GE Healthcare). Polymerase chain reaction (PCR) products were joined by a linker encoding a flexible 15-amino acid chain of (Gly4-Ser)3. The VH-glycine linker–VL complex (scFv) was subcloned between the SfiI and NotI restriction sites of the pCANTAB 5E vector (GE Healthcare).

Recombinant phage antibodies were generated by transforming *E. coli* TG-1 cells with a phagemid vector containing scFv cDNA, followed by infection with an M13-KO7 helper phage. Antigen-reactive phages were isolated by biopanning according to the manufacturer’s instructions. Log-phase TG-1 cells were infected with antigen-reactive phages, and individual antibody-displaying phages from the phage library were screened with ELISAs using recombinant GST-Syt II-C2A bound to microtiter wells. Antigen-reactive phages were visualised using horseradish peroxidase (HRP)-conjugated anti-M13 antibody (1:5000 dilution; GE Healthcare). DNA sequences of scFv-A36 were deposited in the DNA database of Japan under accession number AB472376.

### Construction of expression vectors

Based on the scFv-A36 cDNA sequence, 2 linker primers were designed for PCR amplification in which Kozak and T7 peptide sequences and a BamHI restriction site were introduced into the 5′ flanking region of A36, and a MunI restriction site, hexahistidine residues, and a NotI restriction site were introduced into the 3′ flanking region of A36 using the Kozak (underlined)-T7 peptide (bold)-BamHI (dotted underline) sense linker primer (5′-GCCGCCACCATGGCT**AGCATGACTGGTGGACAGCAAATGGGT**GGATCCTATGCGGCCCAGCCGGCCAGGGCC-3′) and MunI (double underlined)-hexa His (italics)-NotI (underlined) antisense linker primer (5′-CGGCGGCCGCTCA*ATGATGATGATGATGATG*CAATTGCCGTTTTATT-TCCAACTTTGTCCC-3′). The PCR product was directly ligated into the pGEM-T-easy cloning vector (Promega; termed pGEM-scFv-T7-A36; Fig. 1a). The EGFP fragment was amplified from the pEGFP-C1 vector (Clontech, Mountain View, CA, USA) using PCR and the BamHI (dotted underlined) sense linker primer (5′-GCGGATCCATGGTGAGCAAGGGCGAGGAG-3′) and the BglII (double underlined) antisense linker primer (5′-CGAGATCTTCAGAAGAACTCGTCAAGAAGGCG-3′). The digested EGFP fragment was ligated into the BamHI site of pGEM-scFv-T7-A36, yielding the vector pGEM-scFv-GFPA36. The plasmid pET-scFv-GFPA36 was constructed by ligating the BamHI/NotI-digested fragment of pGEM-scFv-GFPA36 into the BamHI and NotI sites of a modified pET3a (M. Fukuda, unpublished data) *E. coli* expression vector (Novagen, Madison, WI, USA). For transient expression of scFv-GFPA36 driven by a cytomegalovirus promoter in mammalian cells, the NotI fragment of pGEM-scFv-GFPA36 was ligated into the pIRES vector (Invitrogen), yielding pIRES-scFv-GFPA36. For the construction of scFv-A36 mutants, a DNA fragment, including the CDR1 and CDR3 regions of the heavy chain of A36, was amplified using PCR and the following degenerate primers: HindIII (underlined) sense linker primer (5′-GCAAAGCTTCTGGCTTCNNNNNNNNNNNNNNNNNNNNNNNNTGGGTGAAGCAGAGGCCTGCACAGG-3′) and BstEII (double underlined) antisense linker primer (5′-GGAGACGGTGACCGTGGTCCCTTGGCCCCANNNNNNNNNNNNNNNNNNNNNNNNAGCACAGTAATAGACGGCAGTGTCCTCAG-3’), in which N is A, C, G, or T (equimolar). The CDR1 and CDR3 mutant fragments were digested with HindIII and BstEII and ligated into the corresponding sites in the parental A36 vector. From these DNA fragments, a mutant scFv-displaying phage library was generated as described above. Multiple alignments of scFv amino-acid sequences were made using CLUSTALW v.2.1^57^. The 3 × Flag tag- (DYKDHDGDYKDHDIDYKDDDDK; Sigma-Aldrich) and HA tag (YPYDVPDYA)-fused scFv constructs (s3Flag-scFv-HA) were synthesised and codon-optimised for expression in mice using Genscript. The s3Flag-fused scFv constructs without the HA tag (s3Flag-scFv) were created using the s3Flag-scFv-HA constructs as a temperate for PCR with the following primers: T7 sense primer (5′-TAATACGACTCACTATAGGG-3′), A36-delHA antisense primer (5′-GGCGAATTCAGAGCTGTCTCTTGATTTCGAGTTTAG-3′), M4-delHA antisense primer (5′-GGCGAATTCAGAGCTGCCGCTTGATTTCGAGTTTAGTCC-3′), and Y13-259-delHA antisense primer (5′-GGCGAATTCTCATTTGATTTCCAGTTTTGTCCCAGC-3′).

For transient expression in mammalian cells, ScFv fragments were cloned into the pEF-BOS vector (a gift from Shigekazu Nagata)^60^. The fragments were cloned into the pET3a vector for expression of STAND proteins in *E. coli* BL21 cells and into lentiviral and/or AAV vectors. For purification of the GST-fused SNARE domain of syntaxin1 (GST-Syx1-SNARE), a DNA fragment corresponding to the SNARE region (amino-acid residues 162–265) of rat syntaxin1B was cloned into the pGEX-4T-3 vector (GE Healthcare) using PCR and the following primers: GS sense primer (5′-CGCGAATTCCGAAGAACTAGAAGACATGTTGG-3′) and GS antisense primer (5′-GCGGAATTCTCAAATTTTCTTCCTCCTGGCC-3′). GST-fused Syt I/II-C2A or C2B was prepared with Glutathione-Sepharose 4B (GE Healthcare). To construct ER-targeted scFv (ER-scFv-A36), a DNA fragment was synthesised by Eurofins; for both the ER-targeting sequence that corresponds to the N-terminal (17 amino acids of mouse calreticulin) and ER-retention signal, KDEL was fused to the N-terminus or C-terminus of scFv-A36, respectively^61^. The DNA fragment of ER-scFv-A36 was cloned into the pEF-BOS vector. To confirm its localisation in the ER, ER-targeted red fluorescent protein (dsRed1, Clontech) (RFP-ER, a gift from Hiroko Bannai)^61^ was co-transfected with an ER-scFv-A36-expressing vector.

### Transfection

HEK293T, HeLa-S3, COS-7, and MIA PaCa-2 cells were cultured with DMEM supplemented with 10% FBS. With the exception of MIA PaCa-2, the cells were transfected with expression vectors using Lipofectamine 2000 (Invitrogen) according to the manufacturer’s instructions and analysed using immunocytochemistry 1 day later. For immunocytochemical detection of cytoplasmic intrabodies, MIA PaCa-2 cells (4 × 10^3^) were seeded on the glass holes (diameter: 12 mm) of 35-mm glass-bottomed dishes (Asahi Techno Glass Corporation); the following day, the cells were infected with lentiviral vectors (2.752 × 10^7^ IU). They were analysed using immunocytochemistry 4 days later.

### Purification of recombinant scFv intrabodies

*E. coli* cells (strain: BL21 [DE3], Novagen) were co-transformed with a pET3a vector (Novagen) expressing scFv-GFPA36 and thioredoxin (Trx)-expressing pT-Trx vector to enhance the cytoplasmic expression of foreign proteins in the cells^62^. The expression of scFv-GFPA36 was induced with 0.1 mM isopropyl-1-thio-β-d-galactopyranoside (IPTG) for 4 h at 30 °C. To purify scFv-GFPA36 or scFv-T7-A36 under non-denaturing conditions, cells were resuspended in phosphate-buffered saline (PBS) buffer containing 100 μM phenylmethylsulfonyl fluoride (PMSF) and sonicated on ice, then solubilised for 10 min at 4 °C by adding Triton X-100 (1%). After centrifugation at 4075 × *g* for 10 min at 4 °C and filtration through a 0.45-μm pore filter, the supernatant was subjected to chromatography with Probond nickel-chelating resin (Thermo Fisher Scientific). The resin was washed with PBS buffer containing 0.1% Triton X-100, followed by washing with PBS alone and a low concentration of L-histidine (10 mM). Bound scFv-GFPA36 or scFv-T7-A36 was eluted from the column with PBS buffer containing a high concentration of L-histidine (100 mM), followed by 3 rounds of dialysis against a buffer composed of 20 mM HEPES-KOH (pH 7.2) and 50 mM NaCl. The STAND-A36, Myc-Y13-259 or STAND-Y13-259 was purified under non-denaturing conditions by co-transforming *E. coli* cells (BL21 [DE3]) with a pET3a vector expressing STAND proteins and a pT-Trx vector. Log phase-transformed cells were induced with 0.1 mM IPTG for STAND-A36 and Myc-Y13-259 or 1 mM IPTG for STAND-Y13-259. Cells were collected by centrifugation at 4075 × *g* for 10 min at 4 °C after 3h-IPTG induction and resuspended in a PBS buffer containing 100 μM PMSF. The cell suspension was sonicated on ice and solubilized for 20 min at 4 °C by adding Triton X-100 (1%). After centrifugation at 4075 × *g* for 10 min at 4 °C and filtration through a 0.45-μm pore filter, the supernatant was purified using chromatography using anti-Flag (M2) antibody-conjugated affinity beads (Sigma-Aldrich) for STAND proteins or anti-Myc antibody-conjugated affinity beads (MBL) for Myc-Y13-259. Bound STAND proteins or Myc-Y13-259 was eluted from the column with a buffer composed of 50 mM Tris–HCl (pH 7.4) and 150 mM NaCl and containing 3 × Flag peptide (1 mg/ml) for STAND proteins or Myc peptide (1 mg/ml), then dialysed 5 times against a buffer composed of 20 mM HEPES-KOH (pH 7.2) and 50 mM NaCl for STAND-A36 or 20 mM HEPES-KOH (pH 7.4) and 50 mM NaCl for Myc-Y13-259 and STAND-Y13-259.

### Measurement of antibody affinity using ELISA

Purified GST (0.25 or 2.5 pmol), GST-Syt I-C2A (0.25 pmol), or GST-Kras (2.5 pmol) containing full-length human Kras in PBS buffer were used to coat 12-well strips (Nalge Nunc International, Rochester, NY, USA; #473709) for 16 h at 24 °C. Each well was blocked with 5% skim milk for STAND-A36, scFv-T7-A36, and scFv-GFPA36 or 2% skim milk for STAND-Y13-259 in PBS buffer containing 0.1% Tween 20 for 1 h at 24 °C, followed by incubation with 0.22–36 nM purified STAND-A36, 1.17–1800 nM purified scFv-T7-A36, 3.77–601 nM purified scFv-GFPA36, or 10.35–667.87 nM purified STAND-Y13-259 for 2 h at 24 °C.

The binding was quantified by incubation with mouse anti-Flag (M2) primary antibody (1/1000 dilution; Sigma-Aldrich) or mouse anti-His antibody (1:100 dilution;　Roche Diagnostics, Indianapolis, IN, USA) and HRP-conjugated anti-mouse secondary antibody (1:1000 dilution; Jackson ImmunoResearch). Specific binding was calculated by subtracting the binding of STAND proteins to GST-coated wells. Nonlinear regression of the STAND-protein binding data was performed using the Hill−Langmuir equation: *B* = *B*_max_ × [STAND protein]/[*K*_d_ + [STAND protein]]

where *B* is the concentration of the STAND protein bound to the antigen, *B*_max_ is the number of total binding sites, [STAND protein] is the free STAND concentration, and *K*_d_ is the dissociation constant. *K*_d_ was calculated using Prism v.8.0 software (GraphPad Inc., San Diego, CA, USA).

### Protein stability assay

The fluorescent dye PSA (PSA200K; ProFoldin) binds to hydrophobic protein surfaces and generates fluorescence at 610 nm. During heating, the fluorescence intensity increases because the hydrophobic region of unfolded, denatured proteins is more exposed on their surface than that of native proteins, and the unfolded proteins have a higher fluorescence intensity. By heating the native proteins, a thermal folding curve can be observed and the protein’s half-life (*t*_1/2_) can be calculated and used to evaluate protein stability^23^.

The stability of the cytoplasmic antibody proteins (STAND-A36: 0.0729 mg/ml; scFv-GFPA36: 0.1 mg/ml; scFv-T7-A36: 0.086 mg/ml) in 20 mM Tris–HCl buffer (pH 8.0) containing 300 mM NaCl, 1 mM dithiothreitol (DTT), and 10% glycerol was monitored with a dye-binding assay. The PSA dye was used according to the manufacturer’s instructions, at 80 °C, with excitation at 550 nm and emission monitored at 610 nm using a spectrofluorometer (Varioskan; Thermo Scientific). The half-lives of the proteins were calculated using the same curve-fitting formula used for the binding *K*_d_, with Prism v.8.0 software (GraphPad Inc.).

### Immunofluorescence analysis

Immunocytochemistry was performed according to standard procedures. Briefly, cells were fixed with 4% paraformaldehyde (TAAB, Berkshire, UK) for 20 min at 24 °C, followed by permeabilization with 0.3% TritonX-100 in PBS for 2 min at 24 °C. The cells were then immediately washed once with a blocking solution composed of 1% bovine serum albumin (BSA, IgG- and protease-free; Jackson ImmunoResearch, West Grove, PA, USA) and 0.1% Triton X-100 in PBS, followed by incubation with the blocking solution for 1 h at 24 °C and then with primary antibodies for 2 h at 24 °C. The following primary antibodies were used: anti-HA (1/300 dilution; Sigma-Aldrich), anti-T7 (1/500 dilution; MBL), anti-Multi Ubiquitin (1/100 dilution; MBL). Immunoreactivity was visualised via incubation with Alexa Fluor 488-, Alexa Fluor 555-, or Alexa Fluor 594-labelled secondary antibodies (1:5000 dilution, 1/2000 dilution, 1/5000 dilution; Invitrogen). The percentage of cells with intrabody aggregates was quantified by counting at least 100 cells per dish in each experiment. Data were obtained from 3 independent experiments.

For immunohistochemistry, mouse brains were fixed in 2% paraformaldehyde. Cryosections (16-μm thick) were permeabilised with 0.3% Triton X-100 in PBS for 2 min at 24 °C, blocked with a blocking buffer composed of 5% BSA in PBS for 1 h at 24 °C, and incubated with primary antibodies in a buffer composed of 1% BSA and 0.1% Triton X-100 in PBS for 16 h at 4 °C. Anti-Flag (1: 4000 dilution; Sigma-Aldrich), anti-HA (1: 680 dilution; Sigma-Aldrich, H6908), anti-TH (1: 500 dilution; Abcam, ab76442) or anti-GFP (1: 1000 dilution; MBL) was used as primary antibodies. Immunoreactivity was visualised with Alexa Fluor 488-, Alexa Fluor 555-, or Alexa Fluor 594-labelled secondary antibodies (1:5000 dilution; Invitrogen). Fluorescence-labelled preparations were imaged with a BZ-9000 fluorescence microscope (Keyence, Osaka, Japan). Images of the SNc were obtained using a BZ-9000 microscope with a ×20 objective lens and merged with a BZ image analyser (Keyence). The integrated fluorescence intensity of tyrosine hydroxylase in the SNc and striatum was quantified using Image J v.1.38e software.

### Immunoblot analysis

Flag-tagged full-length mouse Syt I-XI-expressing vectors were used for determining specific binding of scFv-GFPA36^59^. The total homogenate of COS-7 cells transiently expressing Flag-tagged Syt I-XI was subjected to 10% SDS–PAGE and transferred to a polyvinylidene difluoride (PVDF) membrane. Purified scFv-GFPA36 was used as the primary antibody. Immunoreactive bands were visualised using an enhanced chemiluminescence (ECL) detection system (GE Healthcare) with an anti-GFP polyclonal antibody (1:1000 dilution; MBL) and HRP-labelled anti-rabbit antibodies. Equivalent amounts of Flag-tagged Syts loaded on the gel were confirmed by reprobing the blots with anti-Flag antibody (1:500 dilution; Sigma-Aldrich).

Whether purified STAND-A36 recognised mouse Syt I/II in the brain was investigated by isolating mouse brains and homogenising them in a buffer composed of 20 mM HEPES-NaOH (pH 7.4), 150 mM NaCl, 0.5% TritonX-100, and a complete protease inhibitor cocktail (Roche) by shearing with a 23-G, then with a 27-G, needle syringe and agitating for 1 h at 4 °C. After the mixture was sheared again with a 27-G needle syringe, the total homogenate containing 2.5 μg of protein was subjected to Western blotting using purified STAND-A36 as the primary antibody. Immunoreactive bands were visualised using ECL with anti-Flag (1:500 dilution; Sigma-Aldrich) and HRP-labelled anti-mouse antibodies (1:3000 dilution; Jackson ImmunoResearch).

For mouse brains injected with AAV vectors, brain tissue was isolated and homogenised in a buffer composed of 20 mM HEPES-NaOH (pH 7.4), 150 mM NaCl, 0.5% Triton X-100, and complete protease inhibitor cocktail (EDTA-free) and sheared with a 23-G and then by a 27-G needle syringe with agitation for 1 h at 4 °C. After the mixture was sheared 10 more times with a 27-G needle syringe, the total homogenate was subjected to Western blotting using anti-tyrosine hydroxylase (1: 1000 dilution; Millipore), anti-tubulin (1:1000 dilution; Sigma-Aldrich), anti-Syt I (1:100 dilution; Enzo Life Sciences), and anti-Flag antibodies (1:500 dilution; Sigma-Aldrich). To examine the intra-disulfide bond formation of scFvs, the cell lysate or purified STAND-A36, scFv-T7-A36, or scFv-GFPA36 was boiled in SDS-sample buffer in the absence or presence of 2-mercaptoethanol (2-ME) (final 6%) or DTT (final 5 mM) before SDS-PAGE. Uncropped and unprocessed scans of the most important blots are provided in the Source Data file.

### Co-immunoprecipitation assay

COS-7 cells were co-transfected with pIRES-scFv-GFPA36 or pIRES-scFv-GFPM4 and pEF-BOS-Flag-Syt I or control vector (pEF-BOS) using Lipofectamine 2000; 25 h later, the cells were scraped and homogenised in a buffer composed of 50 mM HEPES-KOH (pH 7.2), 250 mM NaCl, and protease inhibitor cocktail (EDTA-free). The homogenate was centrifuged at 1200 × *g* for 5 min at 4 °C, and the supernatant was solubilised by adding Triton X-100 (0.1%) and agitating for 1 h at 4 °C. After centrifugation at 20,400 × *g* for 10 min at 4 °C, the supernatant was transferred to a new tube, and Flag-Syt I or II was immunoprecipitated with the anti-Flag antibody and Dynabeads protein G (Veritas, Mountain View, CA, USA; 10004D) in the presence of 100 μM CaCl_2_. After the beads were washed with a buffer composed of 50 mM HEPES-KOH (pH 7.2), 250 mM NaCl, 100 μM CaCl_2_, 0.1% Triton X100, and a protease inhibitor cocktail (EDTA-free), the bound proteins were eluted with 0.1 M citrate and analysed using Western blotting with anti-Syt I antibody (1: 100 dilution; Stressgen Biotechnologies) and an HRP-labelled anti-mouse-IgG light-chain-specific secondary antibody (1:5000 dilution; Jackson ImmunoResearch) to detect Flag-Syt I or an HRP-labelled anti-T7 antibody (1:5000 dilution; Millipore) to detect co-immunoprecipitated scFv-GFPA36.

The interaction between Syt I and intracellular STAND-A36 was assessed by co-transfecting 293 T cells with pEF-BOS-Syt I and a pEF-BOS-STAND-A36 or pEF-BOS-STAND-M4 vector using Lipofectamine 2000. After 25 h, the cells were scraped, lysed in a buffer composed of 20 mM HEPES-NaOH (pH 7.4), 150 mM NaCl, 0.5% Triton X-100, and protease inhibitor (EDTA-free) for 1 h, then sheared with a 27-G needle syringe. The homogenate was centrifuged at 17,600 × *g* for 10 min at 4 °C, and the supernatant was transferred to a new tube containing CaCl_2_ (500 μM) and immunoprecipitated with anti-HA agarose (Sigma-Aldrich). After the beads were washed with lysis buffer containing 500 μM CaCl_2_, they were boiled in 1 × SDS sample buffer for 3 min. After centrifugation at 11,300 × *g* for 30 s, the supernatant was used for Western blotting.

Co-immunoprecipitated Syt I was detected with anti-Syt I primary antibody (1: 100 dilution; Enzo Life Sciences) and HRP-labelled anti-mouse-IgG light-chain-specific secondary antibody (1: 5000; Jackson ImmunoResearch). Blots were also probed with an anti-Flag (M2) primary antibody (1: 400 dilution; Sigma-Aldrich) and an HRP-labelled anti-mouse-IgG light-chain-specific secondary antibody (1: 3000 dilution; Jackson ImmunoResearch) to ensure that scFv proteins were precipitated. The inhibitory effect of STAND-A36 on the interaction of Syt I with syntaxin1 was measured by subjecting the above cell lysates to GST pulldown using GST-Syx1-SNARE. The cell lysate (200 μg of protein) was mixed with 2 μl of anti-GST antibody (GE Healthcare; 27-4577-01), Dynabeads protein G, and 2 μg GST or GST-Syx1-SNARE in the presence of 500 μM CaCl_2_. The mixture was agitated for 1 h at 4 °C, then washed with a buffer composed of 20 mM HEPES-NaOH (pH 7.4), 150 mM NaCl, 0.5% Triton X-100, protease inhibitor (EDTA-free), and 500 μM CaCl_2_. Immunoreactivity was visualised using ECL and quantified using ImageJ v.1.38e software.

The intracellular interaction of STAND proteins with endogenous Kras was investigated by performing immunoprecipitation^63^. MIA PaCa-2 cells (4.4 × 10^5^) were seeded on 6-cm culture dishes, then infected with lentiviral vectors expressing STAND proteins (STAND-Y13-259: 4.99 × 10^7^ IU, STAND-A36: 7.7 × 10^7^ IU). After 2 days, cell pellets were resuspended in an immunoprecipitation buffer composed of 20 mM Tris–HCl (pH 7.4), 100 mM NaCl, 1% Triton X-100, 5 mM MgCl_2_, 0.5% deoxycholate, and a protease inhibitor cocktail (EDTA-free), followed by agitation for 1 h at 4 °C. After centrifugation at 17,300 × *g* at 4 °C, the supernatant (350 μg of protein) was incubated with 5.5 μg of rabbit anti-HA antibody (Sigma-Aldrich; H6908) and Dynabeads protein G for 2 h at 4 °C. After the mixture was washed 5 times with a buffer composed of 50 mM HEPES-KOH (pH 7.5), 100 mM NaCl, 1% Triton X-100, 0.5% deoxycholate, 0.1% SDS, 1 mM EDTA, and a protease inhibitor cocktail (EDTA-free), bound proteins were eluted with 0.1 M citrate and subjected to Western blotting with an anti-Kras antibody (1:250 dilution; Santa Cruz Biotechnology) or anti-Flag (M2) antibody (1:500 dilution; Sigma-Aldrich). Immunoreactivity was visualised using an HRP-labelled anti-mouse-IgG light-chain-specific secondary antibody (1:3000 dilution; Jackson ImmunoResearch).

For the GST-pulldown assay using GST-Raf1-RBD (Merk), Kras- and STAND-Y13-259-expressing vectors were co-transfected into 293 T cells using Lipofectamine 2000; 25 h later, the cells were scraped and lysed in a buffer composed of 20 mM Tris–HCl (pH 7.4), 100 mM NaCl, 1% Triton X-100, 5 mM MgCl_2_, 0.5% deoxycholate, and protease inhibitor (EDTA-free) for 1 h. The homogenate was centrifuged at 17,600 × *g* for 30 min at 4 °C, and the supernatant was subjected to GST pulldown using GST-Raf1-RBD-agarose. The cell lysate (1 mg of protein) was mixed with 12.15 μg GST- or 20 μg GST-Raf1-RBD-agarose (50% slurry). The mixture was agitated for 2 h at 4 °C, then washed with a buffer composed of 50 mM HEPES-KOH (pH 7.5), 100 mM NaCl, 1% Triton X-100, 0.5% deoxycholate, 0.1% SDS, 1 mM EDTA, and protease inhibitor (EDTA-free). Precipitates were subjected to Western blot analysis with an anti-Kras antibody (1:250 dilution; Santa Cruz Biotechnology) or anti-Flag (M2) antibody (1:500 dilution; Sigma-Aldrich). Immunoreactivity was visualised using an HRP-labelled anti-mouse-IgG light-chain-specific secondary antibody (1:3000 dilution; Jackson ImmunoResearch). Immunoreactivity was visualised using ECL and quantified using ImageJ v.1.38e software.

### Production of AAV vectors

AAV9/3 vector plasmids contained the rat synapsin I promoter (SynIp), followed by the inverted cDNA of interest between the double loxP sequence (loxP-lox2722) derived from the pAAV-Ef1a-DIO eNpHR 3.0-EYFP vector (a gift from Karl Deisseroth; Addgene, Cambridge, MA, USA; #26966)^64^, a 241 bp fragment (FrgH) containing nucleotides 2519–2760 derived from rat tau 3′-UTR^65^, a woodchuck hepatitis virus posttranscriptional regulatory element (WPRE), and a simian virus 40 polyadenylation signal sequence (SV40pA) between the inverted terminal repeats of the AAV3 genome^66^. The AAV9 viral protein (vp) cDNA was synthesised with substitutions of thymidine for adenine at positions 1337 and 2192. These substitutions introduced amino acid changes from tyrosine to phenylalanine at positions 446 and 731 (Y446F and Y731F)^66^. Recombinant AAV9/3 vectors were produced via transient transfection of HEK293 cells using the vector plasmid, an AAV3 replication (rep) and AAV9 vp expression plasmid, and the adenoviral helper plasmid pHelper (Agilent Technologies, Santa Clara, CA, USA)^66^, yielding the vectors AAV9/3-SynIp-DIO-STAND-A36 or AAV9/3-SynIp-DIO-STAND-M4. Recombinant viruses were purified on 2 sequential continuous CsCl gradients, and viral titres were determined using quantitative PCR. The AAV1 vector plasmids contained SynIp, followed by the cDNA of interest, FrgH, WPRE, and SV40pA between the inverted terminal repeats of the AAV1 genome. Recombinant AAV1 vectors were produced as described above, yielding the AAV1-SynIp-scFv-GFPA36 or M4 vectors.

### Production of lentiviral particles

To obtain lentiviral particles expressing STAND-Y13-259, STAND-A36, or Myc-Y13-259, we constructed a pLenti vector containing the EF promoter (pLenti-pEF vector) in which the EF promoter fragment derived from the pEF-BOS vector was cloned into the XbaI and EcoRI sites of pLenti-hSyn-eNpHR 3.0-EYFP (a gift from Karl Deisseroth; Addgene; #26775)^64^. STAND protein-coding DNA fragments were cloned into the EcoRI site of pLenti-EFp, yielding pLenti-EFp-STAND-Y13-259, pLenti-EFp-STAND-A36, and pLenti-EFp-Myc-Y13-259, respectively. Recombinant lentiviral particles were generated by co-transfecting 293 T cells with pLenti-EFp-scFv along with psPAX2 (Addgene; #12260) and pMD2.G (a gift from Didier Trono; Addgene; #12259). After 2 days, the supernatant was centrifuged at 402 × *g* on an LC-121 centrifuge with a TS-7 rotor (TOMY, Tokyo, Japan) at room temperature to remove cell debris. After the supernatant was filtered through a 0.45-μm filter, lentiviral particles were concentrated with PEG-it Virus Precipitation Solution (System Biosciences, Palo Alto, CA, USA) according to the recommended protocol. The precipitate was resuspended in PBS, and the viral titre was determined using a quantitative PCR Lentivirus Titration (Titre) kit (Applied Biological Materials, Richmond, BC, Canada) according to the manufacturer’s recommendations.

### Stereotactic injection of AAV vectors

Littermates of male DAT-Cre (+/−) mice were randomly assigned to experimental groups. Male wild-type B6 or DAT-Cre (+/−) mice (8 weeks old) were anesthetised with isoflurane (Escain Mylan Co., Osaka, Japan) delivered via a small-animal anesthetiser (MK-A110; Muromachi Kikai Co., Tokyo, Japan) and placed into a stereotaxic frame (Stereotaxic Just for Mouse; Muromachi). During surgery, anaesthesia was maintained with 1.5% isoflurane delivered at a rate of 1.5 l/min. A hole was drilled into the skull at the site of injection (Model 1474 Stereotaxic drill; Muromachi). AAV1-SynIp-scFv-GFPA36/M4 (1 × 10^9^ vg/B6 mouse) and AAV9/3-SynIp-DIO-STAND-A36/M4 (5 × 10^9^ vg/DAT-Cre [+/−] mouse) were injected into the SNc of the right hemisphere (coordinates relative to bregma in mm: AP, −3.08; LR, −1.25; DV, −4.5) using a 33-G needle (RN NDL, 33/PT-4/10 mm/S; Hamilton, Reno NV, USA) and a 5 µl syringe (Model 75 RN SYR; Hamilton) controlled using an injection pump (Legato 130; Muromachi) at 0.2 µl min^−1^ for 2 µl. The needle was left in place for 10 min after the injection. Wild-type B6 mice injected with AAV1-SynIp-scFv-GFPA36/M4 were used for immunohistochemistry 31 days later. DAT-Cre (+/−) mice injected with AAV9/3 vectors were used for microdialysis, behaviooral tests, or immunochemical analysis at least 33 days after the injection.

### Measurement of dopamine release

Seven days after infection with AAV vectors, primary dopaminergic neurons were washed twice with 500 μl of a pre-warmed PSS-LK buffer composed of 20 mM HEPES-NaOH (pH 7.4), 140 mM NaCl, 4.7 mM KCl, 2.5 mM CaCl_2_, 1.2 mM MgSO_4_, 1.2 mM KH_2_PO_4_, and 11 mM glucose, followed by incubation with 200 μl of PSS-LK buffer for 5 min at 37 °C. After collecting the buffer, cells were stimulated with 200 μl of PSS-HK buffer composed of 20 mM HEPES-NaOH (pH 7.4), 85 mM NaCl, 60 mM KCl, 2.5 mM CaCl_2_, 1.2 mM MgSO_4_, 1.2 mM KH_2_PO_4_, and 11 mM glucose or a PSS-HK-EGTA buffer (with the same composition as the PSS-HK buffer but with 1.0 mM EGTA added) for 5 min at 37 °C.

Collected supernatants and cell samples were immediately mixed with 10 μl of 0.1 M perchloric acid (PCA) containing 50 μM EDTA-2Na and sonicated for 30 s, incubated on ice for 30 min, then centrifuged at 20,000 × *g* for 15 min at 0 °C. The supernatant (190 μl) was transferred to a new tube and mixed with 1.7 μl of 5 M CH_3_COONa to adjust the pH to around 3.0. After collecting the PSS-HK buffer, cells were lysed with 200 μl of 5 mM PCA containing 2.5 μM EDTA-2Na. The samples (200 μl) were sonicated and mixed with 2.4 μl of 5 M CH_3_COONa, then filtered through a 0.45-μm PVDF micropore filter (Millipore). The filtrate was analysed using high-performance liquid chromatography (HPLC) in conjunction with an electrochemical detection system (ECD300; Eicom)^67^. The amount of DA released from primary cultured dopaminergic neurons exposed to high-KCl concentrations was normalised with the DA content in a low-KCl buffer.

### In vivo microdialysis in freely moving mice

Four to 5 adult male mice were housed in a cage until microdialysis was completed. Mice were anesthetised with isoflurane via a small-animal anesthetizer and placed in a stereotaxic frame. During surgery, anaesthesia was maintained with 1.5% isoflurane delivered at a rate of 1.5 l/min. A hole was created for the implantation of a steel guide cannula (AG-4; Eicom) in the striatum of the right hemisphere (coordinates relative to bregma in mm: AP, + 0.6; LR, −2.0; DV, −2.0), whereas another hole was created to anchor the stabilising screw. The guide cannula and stabilising screw were fixed with dental cement (Unifast3; GC America, Alsip, IL, USA). After the cement had completely dried, a microdialysis probe (A-I-4-02; Eicom) was inserted into the striatum through the guide. Probe implantation was completed 3.5–4.5 h after the start of anaesthesia. Mice were placed on a paper towel (Kimtowel; Nippon Paper Crecia) in a transparent microdialysis cage (20 cm long × 20 cm wide × 21 cm high; light level: 520 lux) and were supplied with water and food.

On the day after implantation surgery, mice were placed on a new paper towel (Comfort200; Nippon Paper Crecia) in a transparent microdialysis cage without food or water. The basal DA release was measured by collecting dialysates between 10:00 and 11:00 on the day after probe-implantation surgery. The probes were continually perfused with Ringer’s solution containing 147 mM NaCl, 4.02 mM KCl, and 2.25 mM CaCl_2_ using a syringe pump (ESP-32; Eicom) at a rate of 1 μl min^−1^. Dialysates were automatically collected at 4 °C at 10-min intervals in plastic microtubes preloaded with 5 μl of 0.1 M CH_3_COOH/250 μM EDTA-2Na using refrigerated fraction collectors (Refrigerated collector 820; Univentor). Dialysates were collected 16.5–17.5 h after probe implantation and stored at 4 °C until the HPLC analysis on the following day. HPLC was performed using an electrochemical detection system (ECD300; Eicom)^67^.

### Biopsy of brain tissue

Brains were collected immediately after decapitation, and 300-μm-thick frozen coronal sections were prepared. Circular tissue punches (diameter: 1.5 mm) were collected from 3 areas of the posterior SNc (AP coordinate: −2.92 mm) and 5 areas of the posterior striatum (AP coordinate: + 1.18 mm) using a disposable biopsy needle (Biopsy Punch; Kai Medical, Solingen, Germany). Samples were homogenised in 0.1 M perchloric acid containing 0.1 mM EDTA and centrifuged for 15 min at 20,000 × *g* and 0 °C. The supernatant was filtered through a 0.22 μm PVDF filter (GV Durapore; Millipore), and the filtrate was analysed using HPLC.

### Behavioural tests

Five adult mice were housed in a cage until behaviour testing was completed. The open-field test was performed using a transparent open-field box (25 cm long × 25 cm wide × 30 cm high; light level: 520 lux at the centre of the field). Mice were transferred from mouse isolators to the testing room 15 min before the open-field test. The room temperature was maintained at 25 °C. Each mouse was placed at the centre of the open-field box, and its horizontal movements were monitored for 60 min with a video camera (Everio GZ-MG730; Victor Company of Japan, Yokohama, Japan). Distance travelled (cm), time spent (s) in the centre area of the field (36% of the field), and moving speed (cm/s) were quantified using ImageJ EP, a modified version of ImageJ (https://imagej.nih.gov/ij/download.html). The number of rearings was manually quantified.

We used a rotarod for mice (MK-610A, Muromachi) equipped with a large rod, 9 cm in diameter (PTRTA9057X5; Muromachi), covered with slippery Nitoflon adhesive tape (no. 903UL, 0.13 mm thick; Nitto Denko, Osaka, Japan; light level: 35 lux on each rod)^68^. Mice were moved from mouse isolators to the testing room 60 min before the test. Before the training session on the first day, the animals were habituated by allowing them to remain on the stationary drum for 3 min. This was repeated every day for 1 min immediately before the session. The rotation was set at a slow speed (5 rpm, 1.4 m/min on the surface). The mice were placed back on the drum immediately after falling, up to 5 times per session. The test was repeated once a day for 5 consecutive days. The latency to fall was manually recorded, and the total latency on the rod before (Day 1) and after (Day 5) training were analysed using a two-way repeated measures analysis-of-variance (ANOVA).

### Cell proliferation assay

The Cell Titer 96 Aqueous One cell proliferation assay (MTS assay; Promega) was used to quantify cell viability. MIA PaCa-2 cells were infected with the lentivirus (6.88 × 10^6^ IU) 1 day after seeding in each well of a 96-well plate (1 × 10^3^ cells/100 μl medium/well). Cell viability was evaluated 4 days after infection. Cell Titer 96 Aqueous One reagent (20 μl) was added to each well using a multipipetter (Multipette Plus; Eppendorf, Hamburg, Germany), and the plates were incubated at 37 °C in 5% CO_2_ for 120 min. The absorbance at 490 nm was measured on a microplate reader (Model 550; Bio-Rad, California, USA). The background was subtracted by measuring the absorbance of the medium without cells in the same 96-well plate.

### Xenografts, lentiviral injection, and intrabody expression analysis

Human tumour xenograft models were developed by subcutaneously injecting human tumour cells grown from culture into 6-week-old male homozygous nude mice (Balb/c nu/nu; Charles River Japan). MIA PaCa-2 cells were cultured in antibiotic-free DMEM (Wako Pure Chemical Industries; #044-29765) supplemented with serum, and cells were collected by centrifugation at 800 × *g* at room temperature. The cells were washed twice and resuspended in serum-free DMEM. The cell suspension was mixed with 10% Matrigel (Dow Corning, Corning, NY, USA; #356234). The mixture (100 μl/1 × 10^6^ cells) was subcutaneously injected into the right posterior flank of nude mice using a 23-G needle and syringe. The needle was left in place for 30 s after injection.

Tumour size was measured using callipers, and the volume was calculated as (π/6) × length × width × width^69^. When the tumour volume was 26.6–66.9 mm^3^, 44 μl of lentiviral particles (5.5 × 10^7^ IU per tumour) were injected into the tumour at 2 sites (22 μl of lentiviral particles per site) via a 0.5 ml syringe with a 29-G needle (Terumo, Tokyo, Japan; SS-05M2913) at 20 µl min^−1^. The needle was left in place for 20 min after injection. Lentiviral particles were diluted with a MIA PaCa-2 cell culture medium conditioned for 4 days, which was prepared by centrifugation at 715 × *g* in a LC-121 centrifuge with a TS-7 rotor for 2 min at room temperature. A lentiviral injection was performed once a week for 4 weeks.

Relative tumour growth was calculated by normalising the tumour volume with the value determined on the day of the first injection. Expression of intrabodies in tumours dissected from mice 24 days after the first injection was analysed using Western blotting. The tumours were cut into small pieces with scissors and treated with a cell-recovery solution (Corning; #354253) containing a 10% protease inhibitor cocktail (1.83 g tumour per 10 ml solution) at 4 °C for 16 h. Tumours were washed twice with ice-cold PBS, then homogenised with a buffer composed of 20 mM Tris–HCl (pH 7.4), 100 mM NaCl, 1% Triton X-100, 5 mM MgCl_2_, 0.5% deoxycholate, and a protease inhibitor cocktail (EDTA-free; 1.83 g tumour per 10 ml buffer). Homogenates were centrifuged at 1600 × *g* at 4 °C for 5 min. The supernatant (100 μg and 10 μg protein for intrabody and actin detection, respectively) was analysed using Western blotting with anti-HA antibody (1/500 dilution; Sigma-Aldrich) to detect intrabodies and an anti-actin antibody (1/1000 dilution; Sigma-Aldrich) to normalise protein expression.

### Protein sequence analysis

The net charge of intrabodies at cytoplasmic pHs (7.4, 7.03, and 6.60) and pIs were calculated from the amino-acid sequence of each intrabody, including peptide tags, using Protein Calculator v.3.4 (http://protcalc.sourceforge.net/), in which pI values are estimated based on the assumption that all amino-acid residues have pKa values equivalent to those of the isolated residues (although this is not valid for a folded protein).

### Quantification and statistical analysis

Data are representative of at least 3 independent experiments. Results are expressed as the mean ± standard error. Data were analysed using Prism v.8.0 software (GraphPad Inc.). After equal variances were verified with the Brown-Forsythe test, groups with equal variance were compared using a two-tailed non-repeated one-way ANOVA (3 or 4 groups), followed by a Tukey’s post-hoc multiple comparison test, Kruskal–Wallis test (3 groups), followed by Dann’s post-hoc multiple comparisons test, or using a two-way repeated measures ANOVA with Tukey’s or Bonferroni’s post-hoc multiple comparison tests. Two groups were compared with the two-tailed Mann Whitney U test. *P* values < 0.05 were considered statistically significant. Sample size (*n* = number of animals or samples per group) was estimated using PS Power and Sample Size Calculation v.3.1.2 software^70^ (http://biostat.mc.vanderbilt.edu/wiki/Main/PowerSampleSize; *α* = 0.05 for 2 groups or 0.0166 for 3 groups, with a statistical power of *β* = 0.8). Animals were allocated to behavioural tests at random. No blinding was used in mice behaviour tests. The statistical tests, *P* values, *F* values, and significance for figures are listed in Supplementary Table 1.

### Reporting summary

Further information on research design is available in the Nature Research Reporting Summary linked to this Article.

## Supplementary information

Supplementary Information

Reporting Summary

## Data Availability

A Reporting Summary for this Article is available as a Supplementary Information file. The authors declare that the source data underlying Figs. 1b, 1d; 2a, b; 3c, h, I; 6a, e, g, h; 7b, c, d, e; 8a–e; 9a, c, e, h; 10c, f; Supplementary Figs. 2a, b; 3; and 6c, were provided as a Source Data file. DNA sequences of scFv-A36 were deposited in DDBJ (https://www.ddbj.nig.ac.jp/index-e.html). The other data that support the findings of this study including unprocessed scans of the important blots and gels are available within the Article, its Supplementary Information files, its Source Data file, and from the corresponding authors upon reasonable request. Unprocessed images of the most important microscopy data including stable expression of STAND proteins were provided in Figs. 4, 5, and Supplementary Fig. 5.

## Source data

Source Data
