# Supplementary material for: An ultra-stable cytoplasmic antibody engineered for in vivo applications
Source: Nat Commun. 2020 Jan 17;11:336. doi: 10.1038/s41467-019-13654-9 (PMC6969036; doi:10.1038/s41467-019-13654-9)
Supplement: Supplementary file 1 — Supplementary Information [file 41467_2019_13654_MOESM1_ESM.pdf]

An ultra-stable cytoplasmic antibody engineered for *in vivo* applications

Kabayama *et al*.

Supplementary Information (8 figures, 3 tables)

Supplementary Figure 1

a

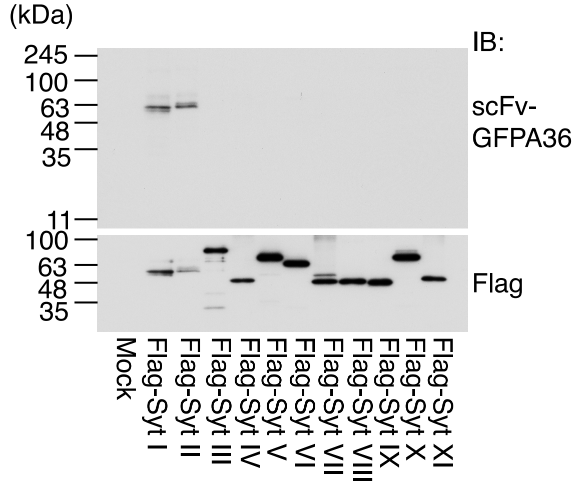

b

5'-degenerate primer

CDR1

CDR2

```
          1
scFv-A36: QVQLQQSGAELVRPGALVKLSCKASGFNIEDYYLHWVKQRPAQGLEWIGGIDPENGIAIYDSKFQGKASI 70
scFv-1:   EVQLQQSGAELVRPGALVKLSCKASGFNIKDYYMHWVKQRPEQGLELIGWIDPENGNTIYDPKFQDKASI 70
scFv-2:   EVKLQQSGAELVRPGALVKLSCKVSGFNIKDYYMNWVKQRPEQGLEWIGWIDPENGNTIYDPKFQGKASI 70
scFv-3:   EVQLQQSGAELVRPGALVKLSCKASGFNIKDNYIHWVRQRPEQGLEWIGRIDPENGYSIYDPKFQGKASI 70
scFv-4:   EVQLQQSGAELVKPGASVKLSCTASGFNIKDTYMHWVKQRPEQGLEWIGRIDPANGNTKYDPKFQGKATI 70
          :*:*********:*** *****..*****:* *::**:*** **** ** *** ** : **.***.**:*
scFv-M4:                             VVVSTTGN
```

3'-degenerate primer

CDR3

```
scFv-A36: TADTSSNTAYLQLSSLTSEDTAVYYCATS---EDSFDVWGQGTTVTVSP 116
scFv-1:   TADTSSNTAYLQLSSLTSEDTAVYYCARD--TAAYFDYWGQGTTLTVSS 117
scFv-2:   TADTSSNTAYLQLSSLTSEDTAVYYCASG--GNPW--YWGQGTTLTVST 115
scFv-3:   TADTSSNTAYLQFSSLTSEDTAVYYCAKDDYYRVGFAYWGQGTLVTVS- 118
scFv-4:   TADTSSNTAYLQLSSLTSEDTAVYYCAH---GRGYFDVWGAGTTVTVSS 116
          ************:**************           ** **  :***
scFv-M4:                             CT   MAGTVE
```

c

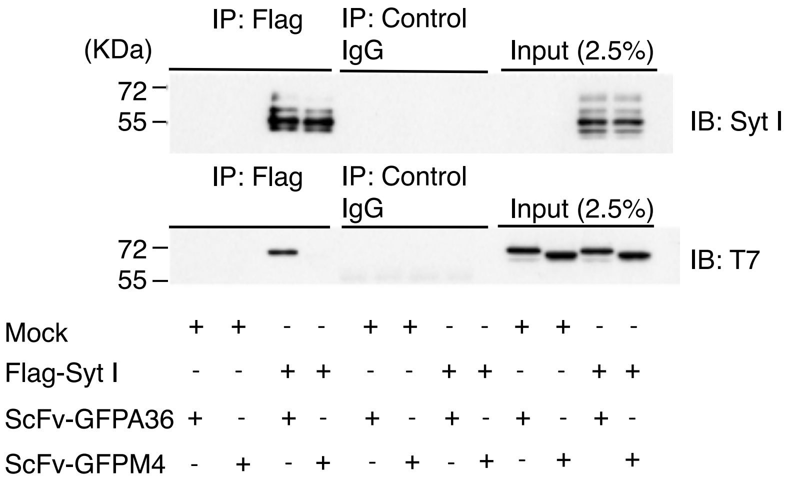

Supplementary Figure 1.

Isolation of scFv-A36 specifically binding to the C2A domain of Syt I/II. (A) Western blot analysis of COS-7 cells expressing Flag-tagged full-length Syt I–XI using purified scFv-GFPA36 as the primary antibody; immunoreactive bands were visualised with anti-GFP antibody and HRP-labelled anti-rabbit antibodies (upper panel). Blots were reprobed with anti-Flag antibody (lower panel). (B) Multiple alignments of amino-acid sequences of VH of 6 scFvs: ScFv-A36 (accession no. AB472376), ScFv-1 (anti-tissue factor D3; accession no. 1K6Q_H), ScFv-2 (anti-white spot syndrome virus; accession no. AAY88907), ScFv-3 (anti-S antigen of hepatitis B virus; accession no. AAD34865), and ScFv-4 (immunoglobulin heavy chain; accession no. AAA16587), and scFv-A36 mutant (termed scFv-M4). Residues identical to A36 in CDRs are shown in red letters. Asterisks indicate amino-acid residues conserved among the 6 scFv proteins. Note that only the amino-acid sequences of CDR1 and CDR3 are shown for scFv-M4. Nucleotide sequences of CDR1 and CDR3 were mutated by PCR with degenerate primers (arrows). (C) Intracellular binding of ScFv-GFPA36, but not of scFv-GFPM4, with Flag-Syt I expressed in COS7 cells. Immunoprecipitates obtained with anti-Flag (M2) antibody were analysed using western blotting with anti-Syt I (SYA148) antibody and HRP-labelled anti-T7 antibody to detect scFv proteins. The predicted molecular weight of ScFv-GFPM4 is slightly less than that of scFv-GFPA36 (A36: 55733.82; M4: 55355.63). In addition, CDR1 and CDR3 of A36 are relatively acidic and residue-rich (5 in 16 residues), while those of M4 are not (1 in 16 residues). The binding of SDS to acidic or basic amino-acid residues is relatively week compared to other amino acids, affecting the migration distance of acidic or basic residue-rich proteins (e.g., histone proteins) in SDS-PAGE. This raises the possibility that the difference in net negative charge of CDR1 and CDR3 of STAND-A36 (–5.1 at pH 7.4) and STAND-M4 (–1.3 at pH 7.4) might affect their migration in SDS-PAGE.

Supplementary Figure 2

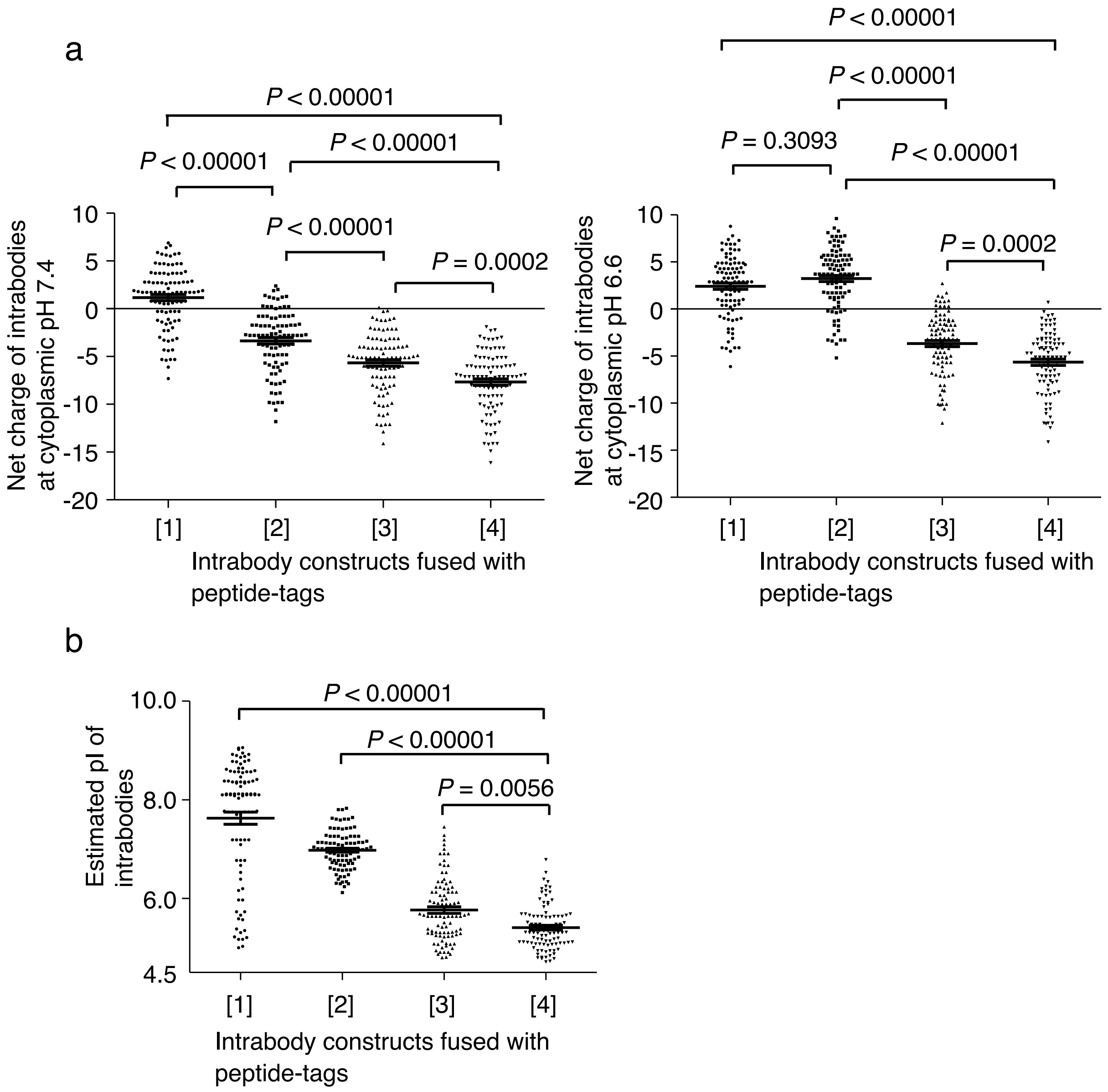

Supplementary Figure 2.
Comparison of the net charge of 94 scFv proteins fused with different peptide tags. (a) Net charge of 94 scFv proteins obtained using a BLAST search for scFv-A36 (Supplementary Table 2) at cytoplasmic pH 7.4 (left panel) and pH 6.6 (right panel). Intrabody constructs fused with peptide tags are described in Fig. 1a. (b) Average pI of 94 scFv proteins fused with indicated peptide tags as described in (a). Statistical analyses were performed using two-tailed one-way ANOVA and Tukey's post hoc multiple comparisons test (Supplementary Table 1). Error bars represent standard error of the mean. Source data are provided as a Source Data file.

Supplementary Figure 3

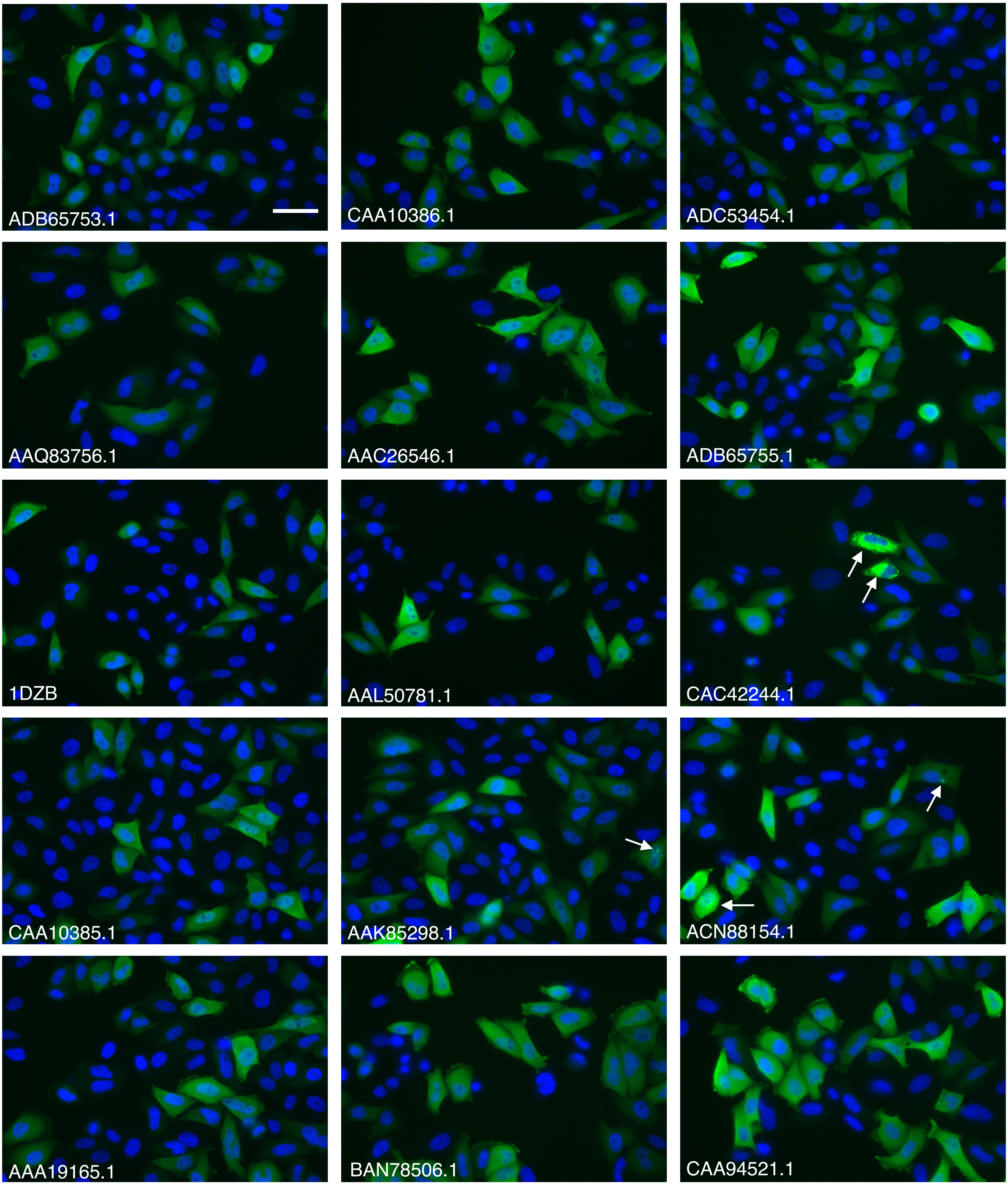

Supplementary Figure 3.
Aggregation rate of scFv proteins in HeLa cells.
Fluorescent images of 15 s3Flag-scFv-HA constructs expressed in HeLa cells. The s3Flag-scFv-HA-expressing vectors were transfected into HeLa cells. The rate of cells with aggregates was measured via immunochemical analysis with anti-HA antibody to detect s3Flag-scFv-HA proteins. Arrows indicate cytoplasmic aggregates of scFv proteins. Scale bars, 50 µm. The accession number of scFvs used is displayed in Supplementary Table 2. Source data are provided as a Source Data file.

Supplementary Figure 4

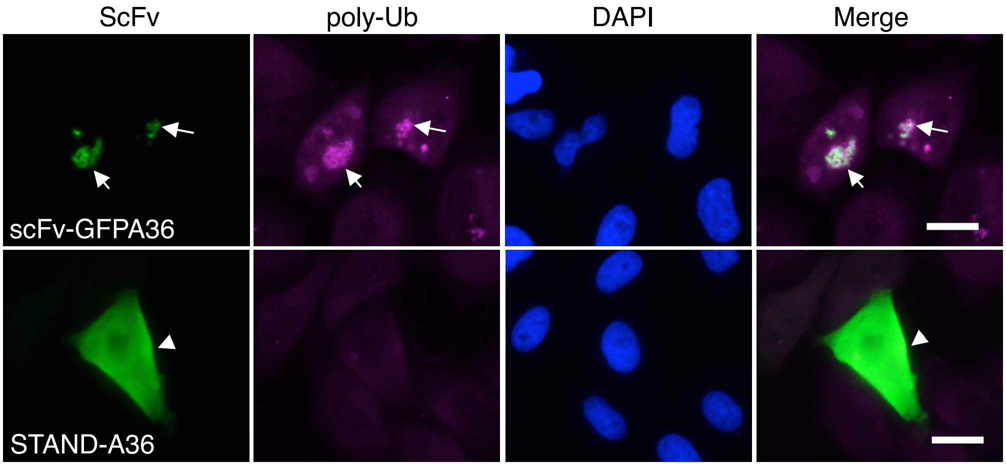

Supplementary Figure 4.
Co-localization of poly-ubiquitin with scFv-GFPA36 aggregates in HeLa cells. Fluorescent images of scFv-GFPA36- (upper panels), or s3Flag-A36-HA- (lower panels) expressing HeLa cells. ScFvs were stained using anti-T7 antibody (upper left, green) or anti-HA antibody (lower left, green). Poly-ubiquitin was detected with anti-multi-ubiquitin antibody (magenta). Arrows indicate cytoplasmic aggregates of scFv-GFPA36 co-localised with poly-ubiquitin. Arrowheads indicate non-poly-ubiquitinated s3Flag-A36-HA stably expressed in the cytoplasm. Scale bar, 20 µm.

Supplementary Figure 5

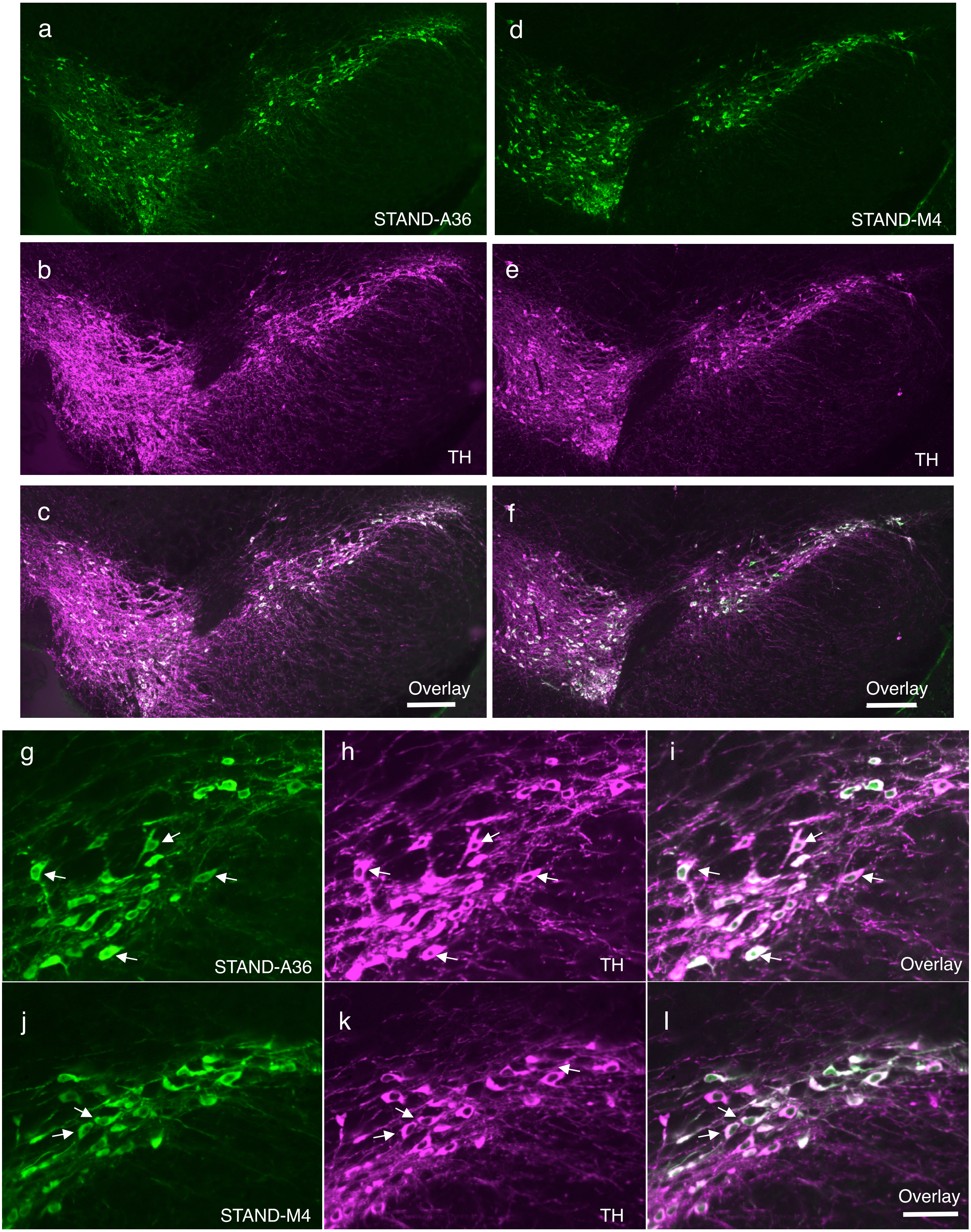

Supplementary Figure 5.
Long-term expression of STAND proteins without aggregation in the SNc.
Fluorescent images of the SNc 6 months after injection with AAV9/3-STAND-A36 (a–c) or AAV9/3-STAND-M4 (d–f). (g–l) Magnified images of the SNc region in a–c or d–f. STAND proteins were stably expressed in the cytoplasm of dopaminergic neurons (arrows). Scale bars, 200 μm (c, f) and 50 μm (l).

Supplementary Figure 6

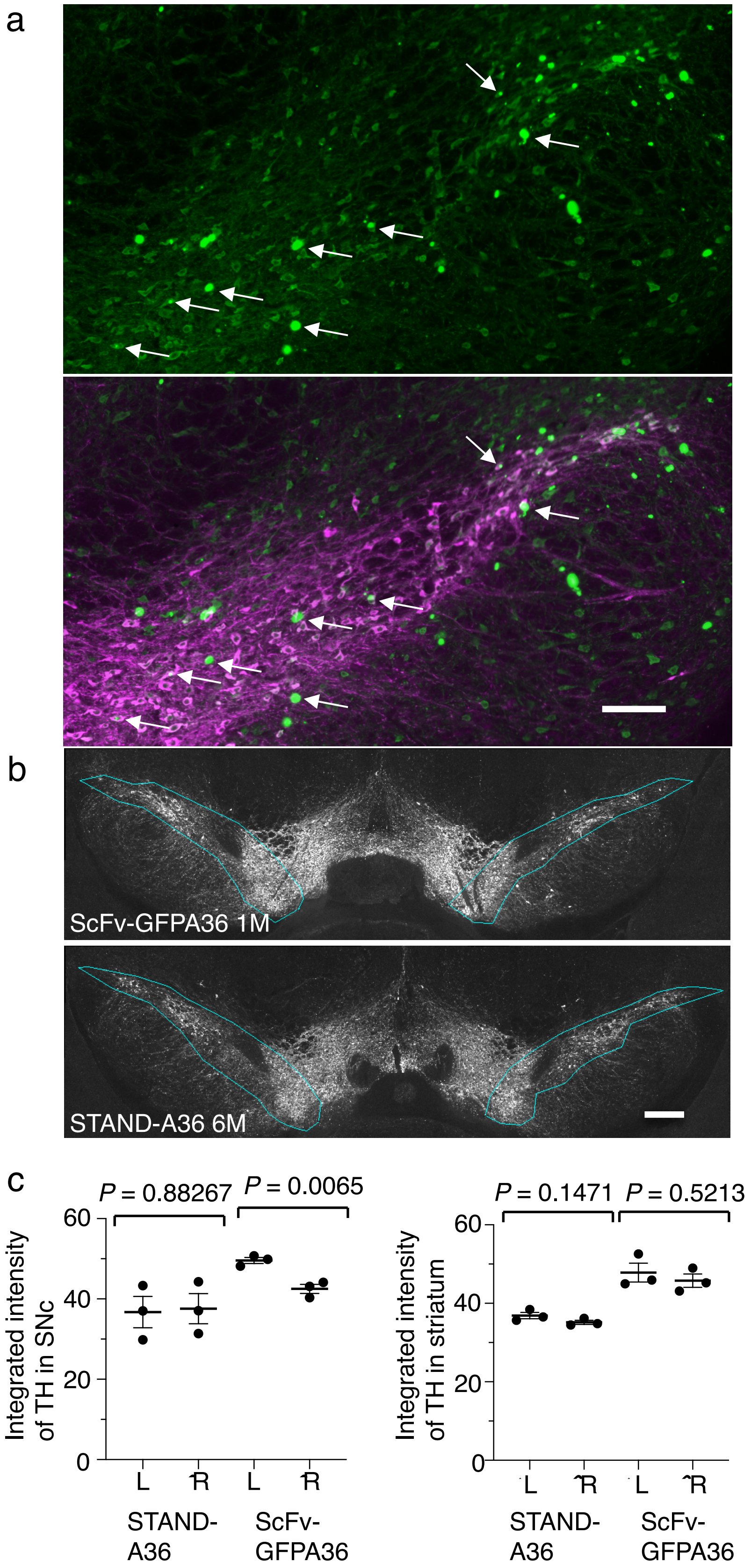

Supplementary Figure 6.

ScFv-GFPA36 causes aggregation and tyrosine hydroxylase reduction the SNc. AAV-scFv-GFPA36 vectors (1×10^9 vg/mouse) were injected into the SNc region of the right cerebral hemisphere. Brains were fixed and analysed using immunohistochemistry 31 days after AAV injection. (a) Fluorescence micrographs of the SNc in the injected right hemisphere are shown. Upper panel: GFP in scFv-GFPA36 (green); lower panel: merged image of scFv-GFPA36 (green) and tyrosine hydroxylase (TH, magenta) expressed in dopaminergic neurons (magenta). ScFv-GFPA36 protein aggregated in the cytoplasm of some neurons (arrows). Scale bar, 100 µm. (b) Fluorescence micrographs of TH in the SNc 31 days (lower, scFv-GFPA36) or 6 months (upper, STAND-A36) after AAV injection. Scale bar, 200 µm. (c) Quantification of fluorescence intensity of TH in SNc (left panel) and striatum (right panel) of the right and left hemisphere. Note that TH expression in the SNc, not the striatum, of the right hemisphere injected with scFv-GFPA36-expressing AAV vectors was 85.74%, a statistically significant decrease when compared with that in the SNc of the non-injected left hemisphere. Statistical analyses were performed using a two-tailed unpaired *t*-test (Supplementary Table 1). Error bars represent standard error of the mean. Source data are provided as a Source Data file.

Supplementary Figure 7

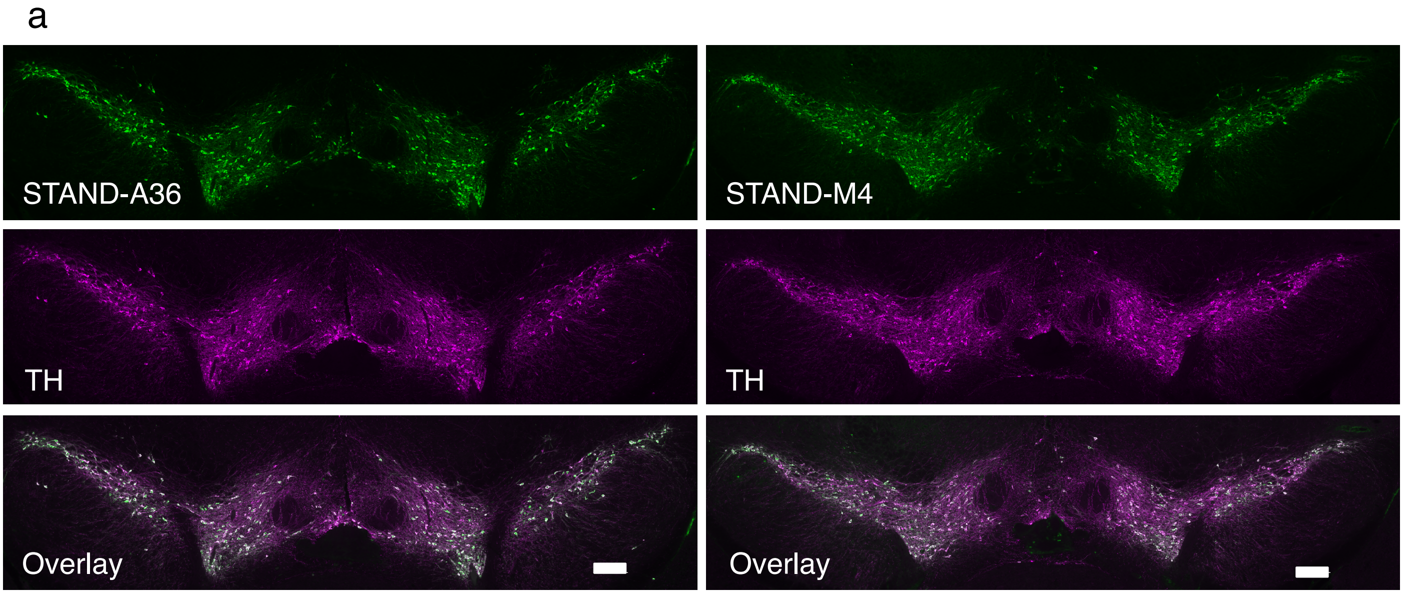

Supplementary Figure 7.
Bilateral expression of STAND-A36 and STAND-M4 in the SNc. (a) Immunohistochemistry of SNc (Green, anti-Flag to detect STAND-A36 [upper left panel] or STAND-M4 [upper right panel]; red, anti-TH antibody to detect dopamine neurons). Scale bar, 200 µm.

Supplementary Figure 8

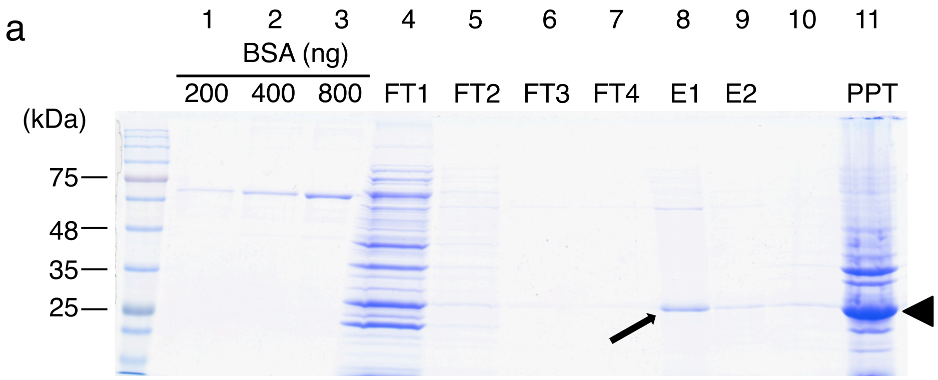

Supplementary Figure 8.
Low efficiency of soluble Myc-Y13-259 purification from *E.coli* cytoplasm. (a) Lanes 1–3, indicate amounts of bovine serum albumin (BSA) as a standard protein; lanes 4–7, washing fractions; lane 8, first eluted fraction with myc peptide; lane 9, second eluted fraction with myc peptide; lane 10, no sample loaded; lane 11, Triton X-100-insoluble fraction (an arrowhead indicates aggregated Myc-Y13-259); Eluted Myc-Y13-259 is indicated by an arrow; molecular weight (kDa) is indicated on the left. Note that the amount of soluble Myc-Y13-259 (lane 8) obtained from 400 mL culture was approximately 10 µg (0.1mg per ml); this is not sufficient to determine the protein stability with PSA, which requires at least 300 µg of proteins for three independent experiments.

Supplementary Table 1

Figure	Comparisons	*F*, *P* values	Post-hocTest	*P* values	Significance
1.d, A36:					
T7, EGFP, His vs. s3Flag	Two-tailed one-way ANOVA	F (2, 6) = 834.2520, P < 0.00001	Tukey's multiple comparisons	< 0.00001	*****
T7, EGFP, His vs. s3Flag, HA	Two-tailed one-way ANOVA	F (2, 6) = 834.2520, P < 0.00001	Tukey's multiple comparisons	< 0.00001	*****
s3Flag vs. s3Flag, HA	Two-tailed one-way ANOVA	F (2, 6) = 834.2520, P < 0.00001	Tukey's multiple comparisons	0.0161	*
1.d, M4:					
T7, EGFP, His vs. s3Flag	Two-tailed one-way ANOVA	F (2, 6) = 180.6460, P < 0.00001	Tukey's post-hoc multiple comparisons	< 0.00001	*****
T7, EGFP, His vs. s3Flag, HA	Two-tailed one-way ANOVA	F (2, 6) = 180.6460, P < 0.00001	Tukey's post-hoc multiple comparisons	< 0.00001	*****
s3Flag vs. s3Flag, HA	Two-tailed one-way ANOVA	F (2, 6) = 180.6460, P < 0.00001	Tukey's post-hoc multiple comparisons	0.0194	*
2.a, pH 6.6	Correlation	Peason r = 0.6181, R squared = 0.3821		0.008179	**
2.a, pH 7.4	Correlation	Peason r = 0.3709, R squared = 0.1376		0.14274	
2.b	Correlation	Peason r = 0.8392, R squared = 0.7042		0.00002542	****
3.h:					
STAND-A36 vs. scFv-GFPA36	Two-tailed one-way ANOVA	F (2, 8) = 15.1284006, P = 0.00191216	Tukey's post-hoc multiple comparisons	0.001816727	**
STAND-A36 vs. scFv-T7-A36	Two-tailed one-way ANOVA	F (2, 8) = 15.1284006, P = 0.00191216	Tukey's post-hoc multiple comparisons	0.014650432	*
scFv-GFPA36 vs. scFv-T7-A36	Two-tailed one-way ANOVA	F (2, 8) = 15.1284006, P = 0.00191216	Tukey's post-hoc multiple comparisons	0.478268323	n.s.
6.a:					
GST vs. GST-Syt I-C2A	Two-tailed one-way ANOVA	F (2, 6) = 9632.99747329107, P = 0.000000000030177	Tukey's post-hoc multiple comparisons	6.6E-14	****
GST vs. GST-Syt I-C2B	Two-tailed one-way ANOVA	F (2, 6) = 9632.99747329107, P = 0.000000000030177	Tukey's post-hoc multiple comparisons	0.220593866	n.s
GST-Syt I-C2A vs. GST-Syt I-C2B	Two-tailed one-way ANOVA	F (2, 6) = 9632.99747329107, P = 0.000000000030177	Tukey's post-hoc multiple comparisons	6.6E-14	****
6.e	Two-tailed Mann Whitney test			0.002164502	**
6.h	Two-tailed unpaired t test			0.031055716	*
7.b:					
M4 vs. A36	Two-tailed one-way ANOVA	F (2, 14) = 9.2457, P = 0.002757	Tukey's post-hoc multiple comparisons	0.0071	**
M4 vs. Non-injected	Two-tailed one-way ANOVA	F (2, 14) = 9.2457, P = 0.002757	Tukey's post-hoc multiple comparisons	0.9352	n.s.
A36 vs. Non-injected	Two-tailed one-way ANOVA	F (2, 14) = 9.2457, P = 0.002757	Tukey's post-hoc multiple comparisons	0.0079	**
7.c:					
Striatum	Two-tailed one-way ANOVA	F (2, 15) = 0.5833, P = 0.5702	Tukey's post-hoc multiple comparisons		n.s.
SN	Two-tailed one-way ANOVA	F (2, 15) = 1.2156, P = 0.3241	Tukey's post-hoc multiple comparisons		n.s.
7.d					
striatum (DOPAC)	Two-tailed one-way ANOVA	F (2, 15) = 0.995351739955074, P = 0.392734666340861			
SN (DOPAC)	Two-tailed one-way ANOVA	F (2, 15) = 1.63394607458641, P = 0.228045313138747			
7.e					
striatum (HVA)	Two-tailed one-way ANOVA	F (2, 15) = 1.07714613351408, P = 0.365500861163008			
SN (HVA)	Two-tailed one-way ANOVA	F (2, 15) = 1.11438894209712, P = 0.353814724336619			
8.a-d					
Total distance	Two-tailed one-way ANOVA	F (2, 19) = 0.688530660451682, P = 0.514415992105662			
Total center time:					
M4 vs. A36	Two-tailed one-way ANOVA	F (2, 19) = 3.79839315463708, P = 0.040952663343881	Tukey's post-hoc multiple comparisons	0.612174388	n.s.
M4 vs. non-injected	Two-tailed one-way ANOVA	F (2, 19) = 3.79839315463708, P = 0.040952663343881	Tukey's post-hoc multiple comparisons	0.223909671	n.s.
A36 vs. non-injected	Two-tailed one-way ANOVA	F (2, 19) = 3.79839315463708, P = 0.040952663343881	Tukey's post-hoc multiple comparisons	0.033782325	*

Moving speed	Two-tailed one-way ANOVA	F (2, 19) = 1.19391403575504, P = 0.324769852481788			
Rearing	Two-tailed one-way ANOVA	F (2, 19) = 0.403672202712696, P = 0.673456897081155			
8.e					
Day 1 vs. Day 5:					
Non-injected	Two-tailed two-way ANOVA	Interaction: F (8, 76) = 2.890, P=0.007267746908046, Time: F (2.771, 52.66) = 30.43, P = 0.000000000039300, Gene: F (2, 19) = 6.208, P = 0.008420081234325	Tukey's post-hoc multiple comparisons	P = 0.007340023845199	**
M4	Two-tailed two-way ANOVA	Interaction: F (8, 76) = 2.890, P=0.007267746908046, Time: F (2.771, 52.66) = 30.43, P = 0.000000000039300, Gene: F (2, 19) = 6.208, P = 0.008420081234325	Tukey's post-hoc multiple comparisons	P = 0.002306060695730	**
A36	Two-tailed two-way ANOVA	Interaction: F (8, 76) = 2.890, P=0.007267746908046, Time: F (2.771, 52.66) = 30.43, P = 0.000000000039300, Gene: F (2, 19) = 6.208, P = 0.008420081234325	Tukey's post-hoc multiple comparisons	P = 0.088553834457845	n.s.
Day 5:					
Non-injected vs. A36	Two-tailed two-way ANOVA	Interaction: F (8, 76) = 2.890, P=0.007267746908046, Time: F (2.771, 52.66) = 30.43, P = 0.000000000039300, Gene: F (2, 19) = 6.208, P = 0.008420081234325	Tukey's post-hoc multiple comparisons	P = 0.0247	*
Non-injected vs. M4	Two-tailed two-way ANOVA	Interaction: F (8, 76) = 2.890, P=0.007267746908046, Time: F (2.771, 52.66) = 30.43, P = 0.000000000039300, Gene: F (2, 19) = 6.208, P = 0.008420081234325	Tukey's post-hoc multiple comparisons	P = 0.9933	n.s.
A36 vs. M4	Two-tailed two-way ANOVA	Interaction: F (8, 76) = 2.890, P=0.007267746908046, Time: F (2.771, 52.66) = 30.43, P = 0.000000000039300, Gene: F (2, 19) = 6.208, P = 0.008420081234325	Tukey's post-hoc multiple comparisons	P = 0.0053	**
Day 1:					
Non-injected vs. A36	Two-tailed one-way ANOVA	Interaction: F (8, 76) = 2.890, P=0.007267746908046, Time: F (2.771, 52.66) = 30.43, P = 0.000000000039300, Gene: F (2, 19) = 6.208, P = 0.008420081234325	Tukey's post-hoc multiple comparisons	P = 0.9630	n.s.
Non-injected vs. M4	Two-tailed one-way ANOVA	Interaction: F (8, 76) = 2.890, P=0.007267746908046, Time: F (2.771, 52.66) = 30.43, P = 0.000000000039300, Gene: F (2, 19) = 6.208, P = 0.008420081234325	Tukey's post-hoc multiple comparisons	P = 0.6099	n.s.
A36 vs. M4	Two-tailed one-way ANOVA	Interaction: F (8, 76) = 2.890, P=0.007267746908046, Time: F (2.771, 52.66) = 30.43, P = 0.000000000039300, Gene: F (2, 19) = 6.208, P = 0.008420081234325	Tukey's post-hoc multiple comparisons	P = 0.4678	n.s.
9.c:					
Myc vs. s3Flag	Two-tailed one-way ANOVA	F (2, 6) = 2110.0416, P < 0.00001	Tukey's post-hoc multiple comparisons	< 0.00001	*****
Myc vs. s3Flag, HA	Two-tailed one-way ANOVA	F (2, 6) = 2110.0416, P < 0.00001	Tukey's post-hoc multiple comparisons	< 0.00001	*****

s3Flag vs. s3Flag, HA	Two-tailed one-way ANOVA	F (2, 6) = 2110.0416, P < 0.00001	Tukey's post-hoc multiple comparisons	0.0141	*
9.h:					
Mock vs. STAND-Y13-259	Two-tailed one-way ANOVA	F (2, 12) = 14.9438, P = 0.0005527	Tukey's post-hoc multiple comparisons	0.0009	***
Mock vs. STAND-A36	Two-tailed one-way ANOVA	F (2, 12) = 14.9438, P = 0.0005527	Tukey's post-hoc multiple comparisons	0.8472	n.s.
STAND-Y13-259 vs. STAND-A36	Two-tailed one-way ANOVA	F (2, 12) = 14.9438, P = 0.0005527	Tukey's post-hoc multiple comparisons	0.0022	**
10.c:					
STAND-Y13-259 vs. Myc-Y13-259	Two-tailed one-way ANOVA	F (3, 8) = 490.4608, P < 0.00001	Tukey's post-hoc multiple comparisons	< 0.00001	*****
STAND-Y13-259 vs. STAND-A36	Two-tailed one-way ANOVA	F (3, 8) = 490.4608, P < 0.00001	Tukey's post-hoc multiple comparisons	< 0.00001	*****
STAND-Y13-259 vs. PBS	Two-tailed one-way ANOVA	F (3, 8) = 490.4608, P < 0.00001	Tukey's post-hoc multiple comparisons	< 0.00001	*****
Myc-Y13-259 vs. PBS	Two-tailed one-way ANOVA	F (3, 8) = 490.4608, P < 0.00001	Tukey's post-hoc multiple comparisons	< 0.00001	*****
STAND-A36 vs. PBS	Two-tailed one-way ANOVA	F (3, 8) = 490.4608, P < 0.00001	Tukey's post-hoc multiple comparisons	0.0641	n.s.
10.f:					
STAND-Y13-259 vs. Myc-Y13-259	Kruskal-Wallis	P = 0.0005310	Dann's post-hoc multiple comparisons	0.0052	**
Myc-Y13-259 vs. STAND-A36	Kruskal-Wallis	P = 0.0005310	Dann's post-hoc multiple comparisons	> 0.99999	n.s.
STAND-Y13-259 vs. STAND-A36	Kruskal-Wallis	P = 0.0005310	Dann's post-hoc multiple comparisons	0.0269	*
Suplementary Figure	Comparisons	*F*, *P* values	Post-hocTest	*P* values	Significance
2.a. pH 7.4:					
No Tag vs. s3Flag, HA	Two-tailed one-way ANOVA	F (3, 372) = 130.8204, P < 0.00001	Tukey's multiple comparisons	< 0.00001	*****
No Tag vs. T7, EGFP, His	Two-tailed one-way ANOVA	F (3, 372) = 130.8204, P < 0.00001	Tukey's multiple comparisons	< 0.00001	*****
T7, EGFP, His vs. s3Flag, HA	Two-tailed one-way ANOVA	F (3, 372) = 130.8204, P < 0.00001	Tukey's multiple comparisons	< 0.00001	*****
T7, EGFP, His vs. s3Flag	Two-tailed one-way ANOVA	F (3, 372) = 130.8204, P < 0.00001	Tukey's multiple comparisons	< 0.00001	*****
s3Flag vs. s3Flag, HA	Two-tailed one-way ANOVA	F (3, 372) = 130.8204, P < 0.00001	Tukey's multiple comparisons	0.0002	***
2.a. pH 6.6:					
No Tag vs. s3Flag, HA	Two-tailed one-way ANOVA	F (3, 372) = 134.3367, P < 0.00001	Tukey's multiple comparisons	< 0.00001	*****
No Tag vs. T7, EGFP, His	Two-tailed one-way ANOVA	F (3, 372) = 134.3367, P < 0.00001	Tukey's multiple comparisons	0.3093	n.s.
T7, EGFP, His vs. s3Flag, HA	Two-tailed one-way ANOVA	F (3, 372) = 134.3367, P < 0.00001	Tukey's multiple comparisons	< 0.00001	*****
T7, EGFP, His vs. s3Flag	Two-tailed one-way ANOVA	F (3, 372) = 134.3367, P < 0.00001	Tukey's multiple comparisons	< 0.00001	*****
s3Flag vs. s3Flag, HA	Two-tailed one-way ANOVA	F (3, 372) = 134.3367, P < 0.00001	Tukey's multiple comparisons	0.0002	***
2.b:					
No Tag vs. s3Flag, HA	Two-tailed one-way ANOVA	F (3, 372) = 184.7457, P < 0.00001	Tukey's multiple comparisons	< 0.00001	*****
T7, EGFP, His vs. s3Flag, HA	Two-tailed one-way ANOVA	F (3, 372) = 184.7457, P < 0.00001	Tukey's multiple comparisons	< 0.00001	*****
s3Flag vs. s3Flag, HA	Two-tailed one-way ANOVA	F (3, 372) = 184.7457, P < 0.00001	Tukey's multiple comparisons	0.0056	**
6.c:					
SN(STAND-A36, R vs. L)	Two-tailed unpaired t test			0.8827	n.s.
SN(scFv-GFPA36, R vs. L)	Two-tailed unpaired t test			0.0065	**
Striatum (STAND-A36, R vs. L)	Two-tailed unpaired t test			0.1471	n.s.
Striatum (scFv-GFPA36, R vs. L)	Two-tailed unpaired t test			0.5213	n.s.

Supplementary Table 1.

The statistical tests, *P* values, *F* values, and significance for Figures and Supplementary Figures

Detailed information for statistical analysis in the manuscript is shown.

Supplementary Table 2

<table>
<tr><td>|ADC53452.1|:3-244</td><td>|AAC26537.1|:1-228</td><td>|ADJ00222.1|:3-248</td></tr>
<tr><td>|ADB65759.1|:3-242</td><td>|CCG26105.1|:20-266</td><td>|ACB88023.1|:1-242</td></tr>
<tr><td>|ADC53454.1|:3-243</td><td>|AAC26550.1|:1-229</td><td>|AAC26530.1|:1-231</td></tr>
<tr><td>|AAC26528.1|:1-228</td><td>|CAA10318.1|:3-248</td><td>|ACS12913.1|:3-240</td></tr>
<tr><td>|ADB65756.1|:3-244</td><td>|S41374:1-247</td><td>|AAL50781.1|:10-234</td></tr>
<tr><td>|ADB65755.1|:3-244</td><td>|AAY88908.1|:3-240</td><td>|AAA83267.1|:1-246</td></tr>
<tr><td>|ADB65753.1|:3-241</td><td>|CAA10385.1|:1-243</td><td>|ACB97617.1|:3-238</td></tr>
<tr><td>|AEK20780.1|:3-237</td><td>|AAC04222.1|:39-276</td><td>|AAP23214.1|:3-247</td></tr>
<tr><td>|AAQ83756.1|:1-248</td><td>|ACZ65029.1|:3-236</td><td>|AAY44382.2|:3-245</td></tr>
<tr><td>|1DZB|A:1-239</td><td>|AAC26540.1|:1-226</td><td>|BAN78505.1|:8-247</td></tr>
<tr><td>|ADB65760.1|:3-238</td><td>|ADN42857.1|:25-265</td><td>|BAA22843.1|:3-242</td></tr>
<tr><td>|CAC14154.1|:2-247</td><td>|ACN88153.1|:3-241</td><td>|AAB05768.1|:2-239</td></tr>
<tr><td>|AEK20779.1|:3-242</td><td>|ACN88155.1|:3-241</td><td>|BAN78507.1|:8-247</td></tr>
<tr><td>|1QOK|A:27-268</td><td>|AAX07566.1|:3-240</td><td>|AF074900_1:3-245</td></tr>
<tr><td>|BAT46707.1|:3-240</td><td>|AAA19165.1|:23-265</td><td>|AAQ83757.1|:1-241</td></tr>
<tr><td>|ADB65757.1|:3-241</td><td>|AAK85297.1|AF402255_1:3-243</td><td>|AAK85298.1|AF402256_1:3-248</td></tr>
<tr><td>|AAY88907.1|:3-240</td><td>|CAD58896.1|:3-233</td><td>|AAK56283.1|:1-239</td></tr>
<tr><td>|ABN79462.1|:2-242</td><td>|ACA49232.1|:3-247</td><td>|AAD33867.1|AF141321_1:3-243</td></tr>
<tr><td>|ADN42858.1|:25-265</td><td>|ADC53451.1|:3-248</td><td>|AAB65160.1|:1-243</td></tr>
<tr><td>|AAF82630.1|:3-243</td><td>|5AAW|A:2-243</td><td>|AAY44384.2|:3-245</td></tr>
<tr><td>|AAD51317.1|:1-241</td><td>|AAC26549.1|:1-232</td><td>|BAN78506.1|:8-247</td></tr>
<tr><td>|ALJ99801.1|:3-251</td><td>|CAA10386.1|:1-249</td><td>|AEX60024.1|:43-282</td></tr>
<tr><td>|AAC26545.1|:1-228</td><td>|ACB97619.1|:3-238</td><td>|AAC25685.1|:1-239</td></tr>
<tr><td>|AAN32896.1|AF488378_1:3-244</td><td>|ADC53455.1|:3-238</td><td>|AAA75173.1|:23-270</td></tr>
<tr><td>|3UYP|A:5-246</td><td>|AAC26546.1|:1-232</td><td>|AEX57225.1|:58-297</td></tr>
<tr><td>|ADB65758.1|:3-241</td><td>|CAA94521.1|:1-248</td><td>|AAL25135.1|AF434672_1:1-237</td></tr>
<tr><td>|AAC52185.1|:3-242</td><td>|AAF82631.1|:3-242</td><td>|AAU10332.1|:22-271</td></tr>
<tr><td>|ADC53456.1|:3-246</td><td>|AAF85943.1|:1-249</td><td>|AHM25305.1|:3-249</td></tr>
<tr><td>|AAU11282.1|:2-240</td><td>|CCG26106.1|:20-263</td><td>|CAC42244.1|:3-244</td></tr>
<tr><td>|A56446:3-242</td><td>|ACV91950.1|:2-239</td><td>|ACN88154.1|:3-241</td></tr>
<tr><td>|CAA82617.1|:1-247</td><td>|AAG44840.1|:1-250</td><td>|ACN87219.1|:3-243</td></tr>
<tr><td></td><td></td><td>JC5322:1-233</td></tr>
</table>

Supplementary Table 2. Accession numbers of ScFv proteins used for in silico analysis.

Amino acid sequences of ScFv proteins were obtained using NCBI BLAST search for scFv-A36.

Supplementary Table 3

REAGENT or RESOURCE	SOURCE	IDENTIFIER
Antibodies		
Rabbit polyclonal anti-T7	MBL International	Cat# PM022;<br>RRID:AB_592788
Mouse monoclonal HRP-conjugated anti-T7	Millipore	Cat#69048-3; RRID: AB_11212778
Mouse monoclonal anti-Flag (M2)	Sigma-Aldrich	Cat# F3165; RRID:AB_259529
Mouse monoclonal anti-Flag (M2) conjugated beads	Sigma-Aldrich	Cat#F2426; RRID: AB_2616449
Mouse monoclonal anti-HA conjugated beads	Sigma-Aldrich	Cat#A2095; RRID: AB_257974
Rabbit polyclonal anti-HA	Sigma-Aldrich	Cat# H6908; RRID:<br>AB_260070
Rabbit polyclonal anti-Myc	MBL	Cat#562; RRID: AB_591105
Rabbit polyclonal anti-GFP	MBL	Cat#598-7; RRID: AB_10597267
Rabbit polyclonal anti-actin	Sigma-Aldrich	Cat#A2066; RRID: AB_476693
Rabbit polyclonal anti-tubulin	Sigma-Aldrich	Cat#T9026; RRID: AB_477593
Mouse monoclonal anti-MAP2	Millipore	Cat#MAB3418; RRID: AB_94856
Mouse monoclonal anti-Syt I	Enzo Life Sciences	Cat#SYA-148F; RRID: AB_311976
Chicken polyclonal anti-TH	Abcam	Cat#ab76442; RRID: AB_1524535
Rabbit polyclonal anti-TH	Millipore	Cat# AB152; RRID:AB_390204
Mouse monoclonal anti-Kras	Santa cruz	Cat# sc-30 RRID:<br>AB_627865
Goat polyclonal anti-rabbit IgG highly cross-absorbed Alexa Fluor 488	Thermo Fisher Scientific	Cat# A-11034 also A11034 RRID:AB_2576217
Goat polyclonal anti-mouse IgG highly cross-absorbed Alexa Fluor 488	Thermo Fisher Scientific	Cat# A-11029 also A11029; RRID:AB_138404
Goat polyclonal anti-mouse IgG highly cross-absorbed Alexa Fluor 594	Thermo Fisher Scientific	Cat# A-11032; RRID:AB_2534091
Goat polyclonal anti-rabbit IgG highly cross-absorbed Alexa Fluor 594	Thermo Fisher Scientific	Cat# A-11037; RRID:AB_2534095
Goat polyclonal anti-chicken IgY Alexa Fluor 594	Thermo Fisher Scientific	Cat# A-11042; RRID:AB_2534099
HRP-anti-mouse IgG Light chain specific	Jackson ImmunoResearch Labs	Cat# 115-035-174; RRID:AB_2338512
HRP-anti-rabbit IgG Light chain specific	Jackson ImmunoResearch Labs	Cat# 211-032-171; RRID:AB_2339149
Murine IgG Control Antibody (mouse serum)	Sigma-Aldrich	Cat# I5381, RRID:AB_1163670
anti-His6 from mouse IgG1	Sigma-Aldrich	Cat# 11922416001, RRID:AB_514486
anti-multi ubiquitin(cloneFK2)	MBL	Cat# D058-3, RRID:AB_592937
Goat anti-mouse (H+L)555	Abcam	Cat# 150118, RRID:AB_2714033
HRP-anti-M13	GE Healthcare	Cat# 27942101, RRID:AB_2616587

Supplementary Table 3.

Antibodies used in this study

The specificity of antibodies used was validated by suppliers, previous studies, or the current study.
