# Supplementary material for: An ultra-stable cytoplasmic antibody engineered for in vivo applications
Source: Nat Commun. 2020 Jan 17;11:336. doi: 10.1038/s41467-019-13654-9 (PMC6969036; doi:10.1038/s41467-019-13654-9)
Supplement: Supplementary file 2 — Reporting Summary [file 41467_2019_13654_MOESM2_ESM.pdf]

nature research

Corresponding author(s): Hiroyuki Kabayama

Reporting Summary

Nature Research wishes to improve the reproducibility of the work that we publish. This form provides structure for consistency and transparency in reporting. For further information on Nature Research policies, see Authors & Referees and the Editorial Policy Checklist.

Statistical parameters

When statistical analyses are reported, confirm that the following items are present in the relevant location (e.g. figure legend, table legend, main text, or Methods section).

n/a	Confirmed	
☐	☒	The exact sample size (*n*) for each experimental group/condition, given as a discrete number and unit of measurement
☐	☒	An indication of whether measurements were taken from distinct samples or whether the same sample was measured repeatedly
☐	☒	The statistical test(s) used AND whether they are one- or two-sided<br>*Only common tests should be described solely by name; describe more complex techniques in the Methods section.*
☐	☒	A description of all covariates tested
☐	☒	A description of any assumptions or corrections, such as tests of normality and adjustment for multiple comparisons
☐	☒	A full description of the statistics including central tendency (e.g. means) or other basic estimates (e.g. regression coefficient) AND variation (e.g. standard deviation) or associated estimates of uncertainty (e.g. confidence intervals)
☐	☒	For null hypothesis testing, the test statistic (e.g. *F*, *t*, *r*) with confidence intervals, effect sizes, degrees of freedom and *P* value noted<br>*Give P values as exact values whenever suitable.*
☒	☐	For Bayesian analysis, information on the choice of priors and Markov chain Monte Carlo settings
☒	☐	For hierarchical and complex designs, identification of the appropriate level for tests and full reporting of outcomes
☒	☐	Estimates of effect sizes (e.g. Cohen's *d*, Pearson's *r*), indicating how they were calculated
☐	☒	Clearly defined error bars<br>*State explicitly what error bars represent (e.g. SD, SE, CI)*

Our web collection on statistics for biologists may be useful.

Software and code

Policy information about availability of computer code

Data collection	Fluorescent images of the cultured cells and brain sections were obtained by a Keyence BZ-9000 image acquisition software.
Data analysis	Fluorescent images of the cultured cells and brain sections were analyzed by a Keyence BZ analyzer software or ImageJ v.1.38e software. GraphPad Prism 8 software was used for statistical analysis, calculation of Kd and T1/2.

For manuscripts utilizing custom algorithms or software that are central to the research but not yet described in published literature, software must be made available to editors/reviewers upon request. We strongly encourage code deposition in a community repository (e.g. GitHub). See the Nature Research guidelines for submitting code & software for further information.

Data

Policy information about availability of data

All manuscripts must include a data availability statement. This statement should provide the following information, where applicable:
- Accession codes, unique identifiers, or web links for publicly available datasets
- A list of figures that have associated raw data
- A description of any restrictions on data availability

A reporting summary for this Article is available as a Supplementary Information file. The authors declare that the data for the graphs are available in the Source Data file. The other data that support the findings of this study are available within the article and its supplementary information files and from the corresponding

author upon reasonable request.

Field-specific reporting

Please select the best fit for your research. If you are not sure, read the appropriate sections before making your selection.

☒ Life sciences ☐ Behavioural & social sciences ☐ Ecological, evolutionary & environmental sciences

For a reference copy of the document with all sections, see nature.com/authors/policies/ReportingSummary-flat.pdf

Life sciences study design

All studies must disclose on these points even when the disclosure is negative.

Sample size	Sample size (n = number of animals or samples per group) was estimated using PS Power and Sample Size Calculation v.3.1.2 software 63 (http://biostat.mc.vanderbilt.edu/wiki/Main/PowerSampleSize; α = 0.05 for two groups or 0.0166 for three groups, with a statistical power of β = 0.8).
Data exclusions	No data were excluded from the analysis in the present study.
Replication	All attempts at replication were successful.
Randomization	Animals in behavior test were allocated at random.
Blinding	Investigators were no binded to study to account for appropriate handling and data acquisition.

Reporting for specific materials, systems and methods

Materials & experimental systems

n/a	Involved in the study	
☒	☐	Unique biological materials
☐	☒	Antibodies
☐	☒	Eukaryotic cell lines
☒	☐	Palaeontology
☐	☒	Animals and other organisms
☒	☐	Human research participants

Methods

n/a	Involved in the study	
☒	☐	ChIP-seq
☒	☐	Flow cytometry
☒	☐	MRI-based neuroimaging

Antibodies

Antibodies used

Mouse monoclonal anti-T7: Millipore, Cat#69522-3, RRID: AB_11211744,
Mouse monoclonal HRP-conjugated anti-T7: Millipore, Cat#69048-3 RRID: AB_11212778,
Mouse monoclonal anti-Flag (M2): Sigma-Aldrich, Cat#P2983, RRID: AB_439685,
Mouse monoclonal anti-Flag (M2) conjugated beads: Sigma-Aldrich, Cat#F2426, RRID: AB_2616449,
Mouse monoclonal anti-HA: Sigma-Aldrich, Cat#A2095, RRID: AB_257974,
Mouse monoclonal anti-HA conjugated beads: Sigma-Aldrich Cat#A2095, RRID: AB_257974,
Rabbit polyclonal anti-HA: Sigma-Aldrich, Cat# H6908, RRID: AB_260070,
Rabbit polyclonal anti-Myc: MBL, Cat#562, RRID: AB_591105,
Goat polyclonal anti-GST: GE Healthcare, Cat#27-4577-01, RRID: AB_771432,
Goat polyclonal HRP-conjugated anti-GST: GE Healthcare, Cat#RPN1236, RRID: AB_771429,
Rabbit polyclonal anti-GFP: MBL, Cat#598-7, RRID: AB_10597267,
Rabbit polyclonal anti-actin: Sigma-Aldrich, Cat#A2066, RRID: AB_476693,
Rabbit polyclonal anti-tubulin: Sigma-Aldrich, Cat#T9026, RRID: AB_477593,
Mouse monoclonal anti-MAP2: Millipore, Cat#MAB3418, RRID: AB_94856,
Mouse monoclonal anti-Syt I: Enzo Life Sciences, Cat#SYA-148F, RRID: AB_311976,
Chicken polyclonal anti-TH: Abcam, Cat#ab76442, RRID: AB_1524535,
Mouse monoclonal anti-Kras: Santa cruz, Cat# sc-30 RRID, AB_627865,
Goat polyclonal anti-rabbit IgG highly cross-absorbed Alexa Fluor 488: Thermo Fisher Scientific, Cat# A-11034 also A11034, RRID:AB_2576217,

	Goat polyclonal anti-mouse IgG highly cross-absorbed Alexa Fluor 488: Thermo Fisher Scientific, Cat# A-11029 also A11029, RRID:AB_138404,<br>HRP-anti-M13: GE Healthcare, Cat# 27942101, RRID:AB_2616587<br>Goat polyclonal anti-mouse IgG highly cross-absorbed Alexa Fluor 594: Thermo Fisher Scientific, Cat# A-11032, RRID:AB_2534091,<br>Goat polyclonal anti-chicken IgY Alexa Fluor 594: Thermo Fisher Scientific, Cat# A-11042, RRID:AB_2534099,<br>HRP-anti-mouse IgG Light chain specific: Jackson ImmunoResearch Labs, Cat# 115-035-174, RRID:AB_2338512,<br>Murine IgG Control Antibody (mouse serum): Sigma-Aldrich, Cat# I5381, RRID:AB_1163670<br>anti-His6 from mouse IgG1: Sigma-Aldrich, Cat# 11922416001, RRID:AB_514486<br>anti-multi ubiquitin(cloneFK2): MBL, Cat# D058-3, RRID:AB_592937<br>Goat anti-mouse (H+L)555 Abcam, Cat# 150118, RRID:AB_2714033
Validation	Specificity of antibodies used was validated by suppliers, previous studies, or the current study.

Eukaryotic cell lines

Policy information about cell lines

Cell line source(s)	HEK293T, HeLa-S3, and COS-7 cells and highly malignant MIA PaCa-2 pancreatic adenocarcinoma cells obtained from RIKEN Bioresource Center Cell Bank (Tsukuba, Japan)
Authentication	We declare that none of the cell lines used were authenticated.
Mycoplasma contamination	RIKEN Bioresource Center Cell Bank confirmed that all cell lines tested negative for mycoplasma contamination.
Commonly misidentified lines (See ICLAC register)	No misidentified lines were used in this study.

Animals and other organisms

Policy information about studies involving animals; ARRIVE guidelines recommended for reporting animal research

Laboratory animals	Balb/c for immunization (female, age: 2 months), Balb/c-nu (male, age: 6 weeks) for xenograft, C57B6/J (male) for AAV injection (age: 2 months) and behavior test (age: 3 months).
Wild animals	This study did not involve wild animals
Field-collected samples	This study did not involve samples collected in the field.
